# No Time to Waste: Transcriptome Study Reveals that Drought Tolerance in Barley May Be Attributed to Stressed-Like Expression Patterns that Exist before the Occurrence of Stress

**DOI:** 10.3389/fpls.2017.02212

**Published:** 2018-01-09

**Authors:** Agnieszka Janiak, Miroslaw Kwasniewski, Marta Sowa, Katarzyna Gajek, Katarzyna Żmuda, Janusz Kościelniak, Iwona Szarejko

**Affiliations:** ^1^Department of Genetics, University of Silesia in Katowice, Katowice, Poland; ^2^Centre for Bioinformatics and Data Analysis, Medical University of Bialystok, Bialystok, Poland; ^3^Department of Plant Anatomy and Cytology, University of Silesia in Katowice, Katowice, Poland; ^4^Department of Plant Physiology, Faculty of Agriculture and Economics, University of Agriculture of Krakow, Kraków, Poland

**Keywords:** barley, drought tolerance, root system, stress, transcriptomics

## Abstract

Plant survival in adverse environmental conditions requires a substantial change in the metabolism, which is reflected by the extensive transcriptome rebuilding upon the occurrence of the stress. Therefore, transcriptomic studies offer an insight into the mechanisms of plant stress responses. Here, we present the results of global gene expression profiling of roots and leaves of two barley genotypes with contrasting ability to cope with drought stress. Our analysis suggests that drought tolerance results from a certain level of transcription of stress-influenced genes that is present even before the onset of drought. Genes that predispose the plant to better drought survival play a role in the regulatory network of gene expression, including several transcription factors, translation regulators and structural components of ribosomes. An important group of genes is involved in signaling mechanisms, with significant contribution of hormone signaling pathways and an interplay between ABA, auxin, ethylene and brassinosteroid homeostasis. Signal transduction in a drought tolerant genotype may be more efficient through the expression of genes required for environmental sensing that are active already during normal water availability and are related to actin filaments and LIM domain proteins, which may function as osmotic biosensors. Better survival of drought may also be attributed to more effective processes of energy generation and more efficient chloroplasts biogenesis. Interestingly, our data suggest that several genes involved in a photosynthesis process are required for the establishment of effective drought response not only in leaves, but also in roots of barley. Thus, we propose a hypothesis that root plastids may turn into the anti-oxidative centers protecting root macromolecules from oxidative damage during drought stress. Specific genes and their potential role in building up a drought-tolerant barley phenotype is extensively discussed with special emphasis on processes that take place in barley roots. When possible, the interconnections between particular factors are emphasized to draw a broader picture of the molecular mechanisms of drought tolerance in barley.

## Introduction

One of the main problems that is addressed by plant science in recent years is related to the mechanisms of plant tolerance to environmental stresses. Climate changes in a longer term and the variable weather patterns in a short term shape the need for better understanding the physiological and molecular background of such tolerance. This understanding provides knowledge on the mechanisms to be targeted in crop breeding programs ensuring the development of new cultivars that are able to produce high yield in changing environmental conditions.

Drought is one of the major factors that negatively impact crop production. The study of extreme weather disasters in the years 1964–2007 and their influence on cereal production shows that drought reduces the national cereal production by 10.1% on average (Lesk et al., 2016). Interestingly, cereal production deficit was higher in more developed countries of North America, Europe and Australasia compared to countries from Asia or Africa. There are several possible explanations of such observation, but one of them points to the fact, that in low-income countries the diversity of crops and management systems is higher than in the developed countries (Lesk et al., 2016). This possibility is well understood by geneticists and breeders who search for new sources of genetic variation, which may be potentially related to higher drought tolerance in crop plants.

The main difficulty in the successful selection of genes and alleles responsible for greater drought tolerance is the complexity of plant response to this type of stress. In general, plant survival strategies under drought may rely on transient responses, such as reduced transpiration or hydrotropism, or on developmental changes leading to deeper root system, reduction of leaf area and adjusted osmotic status allowing to minimize water loss and improve water uptake resulting in the survival of longer periods of drought (Hu and Xiong, 2014). Both, transient response and developmental changes require a substantial rebuilding of plant metabolism and changes in the expression of a high number of genes upon the onset and persistence of drought.

Global transcriptome profiling gives an opportunity to have deeper insight into the complexity of plant response to drought stress on the molecular level. Gene expression studies of various plant species exposed to drought stress point to the importance of several groups of genes, which are regulated by this stress. The first includes drought signaling and transcription regulation. Among them, several calcium-dependent protein kinases, calmodulin and calmodulin-related calcium sensor proteins and protein phosphatases class 2C (PP2C) were detected (Molina et al., 2008; Guo et al., 2009; Ranjan and Sawant, 2015), together with a number of transcription factors (TFs) from various families including DREB, AP2/ERF, NAC, bZIP, MYB/MYC, CAMTA, Alfin-like, Q-type ZFP, or HD-START (Sahoo et al., 2013; Janiak et al., 2016). These signaling proteins, TFs, as well as their downstream targets are usually categorized to ABA-dependent and ABA-independent stress response pathways (Shinozaki and Yamaguchi-Shinozaki, 2007). A prominent role in drought response is given to 9-cis-epoxycarotenoid dioxygenase (NCED), which is the key enzyme in ABA biosynthesis (Iuchi et al., 2001) and to PYR/PYL/RCAR receptors of ABA that are responsible for ABA-dependent stomatal closure (Gonzalez-Guzman et al., 2012). Genes involved in biosynthesis and signaling pathways of other plant hormones, such as auxin, ethylene, jasmonic or salicylic acid, were also identified as differentially expressed under drought (Jakoby et al., 2002; Aimar et al., 2011). Other groups of genes differentially regulated by drought are related to antioxidation processes, osmoprotectant synthesis and various factors from LEA family (Shinozaki and Yamaguchi-Shinozaki, 2007; Talame et al., 2007).

Data on drought-responsive transcriptome profiling in wild and cultivated barley were collected in several experiments based on microarrays or, more recently, RNA-Seq experiments. Most of them concentrated on leaf transcriptomes (Talame et al., 2007; Guo et al., 2009; Bedada et al., 2014; Wehner et al., 2016; Zeng et al., 2016) and several studies analyzed other above-ground organ, such as spikelets, awns, seeds (Abebe et al., 2010; Hubner et al., 2015), or crowns (Svoboda et al., 2016). Only one study was aimed at transcriptome analysis of barley roots in the context of root hairs role in environmental stress sensing (Kwasniewski et al., 2016).

The presented paper aims to fill this gap in the knowledge on the transcriptional changes in roots of barley subjected to water deficit and to compare it with drought response in leaves. We also propose a different point of view on the establishment of drought-tolerant phenotype, which assumes that a certain level of transcription of stress-influenced genes is required even before the onset of drought, for better survival of water deficits.

For this purpose two barley genotypes were used: a European cultivar “Maresi” and a Syrian breeding line Cam/B1/CI08887/CI05761 (referred later as CamB). These two genotypes differ in the level of drought tolerance, namely, CamB genotype being more drought tolerant than the European cultivar (Filek et al., 2015). At seedling stage, after 10-days drought stress, CamB genotype had a higher relative water content (RWC), a smaller reduction of the parameters of photosynthetic activity, a smaller drop in chlorophyll a content, and a higher sugar content than Maresi cultivar. The activity of superoxide dismutase (SOD), catalase (CAT), and peroxidases (POX) in CamB genotype did not change after exposition to the stress, and except for CAT, it was generally lower than in Maresi cultivar (Filek et al., 2015), indicating that CamB experiences less of oxidative stress than Maresi. After drought stress, well-developed chloroplasts were still visible in leaves of CamB genotype and their number was not reduced in a significant way, whereas in Maresi less chloroplasts and of significantly smaller size were noticed (Filek et al., 2016). CamB genotype showed its tolerance to drought also in terms of radicals and metal ions content. It was found that Mn(II) ions were better hydrated and the content of Fe(III) ions was lower in this genotype comparing to Maresi, indicating better water accumulation in CamB and a lower level of reactive oxygen species (ROS) formed in the tolerant genotype (Filek et al., 2016).

In the presented study we have used the second leaf and the whole root system of CamB and Maresi genotypes subjected to 10-days of drought stress for RNA extraction and subsequent hybridization with 4 × 44 K Agilent Barley Gene Expression Arrays. Our data indicate that the drought tolerant genotype has a different initial level of expression of many genes in comparison to the sensitive cultivar, what may be important in building up tolerance to the stress. The possible role of these genes is extensively discussed, with special emphasis on the processes that take place in barley roots.

## Materials and methods

### Plant material and stress treatment

Two barley genotypes were used for the study: a German semidwarf cultivar Maresi and a Syrian breeding line Cam/B1/CI08887//CI05761 (CamB), adapted to dry environments. Grains of both genotypes were sterilized and sown in the pots with a dimension of 37 × 15 × 14 cm (L × W × H). The pots were filled with a mixture of sandy loam and sand (7:2 w/w). In this substrate, a pF range of 2.2–3.0 indicated easily available water and pF > 4.2 was the permanent wilting point, as calculated based on the water retention curve. Initially, 15 seedlings were placed in pots, and after germination, the number of plants was reduced to 10. At this time, the seedlings were transferred to an air-conditioned greenhouse, where the temperature was maintained at 20/17°C (day/night), with the light intensity of 520 μmol(photon)m^−2^ s^−2^. The substrate humidity was determined by monitoring pots weight. Soil drought (3.65%, i.e., pF = 4.0) was applied to plants after the appearance of the 4th leaf and it was continued for 10 days. Plants grown in pots with 11% water content (pF = 2.8) were used as the control. Drought stressed and control plants were characterized on physiological level and the results of these analysis were published by Filek et al. (2015). The whole experiment was carried out in three replicates, where one pot was considered as one biological replication. In transcriptome analysis one replication consisted of material from three plants collected from one pot. Their tissues were bulked together and subsequently homogenized serving as a material for one RNA extraction.

### Material collection and RNA isolation

After 10-days of drought stress the second leaf and the whole root system were collected for RNA extraction from both, drought-treated and control plants. To collect roots, plants were gently removed from the soil, separated from each other and roots were briefly washed in water to remove the soil substrate. The washing time did not exceed 30 s. Leaves and roots were frozen in liquid nitrogen and were subsequently homogenized in a sterile, ice-cold mortar. Homogenized tissue was divided into smaller portions suitable for RNA extraction. Total RNA was extracted using RNeasy Plant Mini kit (Qiagen, Hilden, Germany), according to the manufacturer's instructions. Extracted RNA was additionally purified using precipitation in 1 M lithium chloride, and each RNA precipitate was then dissolved in 15 μl of nuclease-free H_2_O. The yield and purity of the RNA was determined using a NanoDrop ND-1000 spectrophotometer (NanoDrop Technologies, USA). The integrity of the RNA was checked using denaturation agarose gel electrophoresis using pre-cast gels and FlashGel RNA System (Lonza, Switzerland). One RNA extract per each replicate was used for subsequent microarray hybridization and one hybridization per each treatment and cultivar was done.

### Preparation of microarrays and microarray data analysis

The synthesis, labeling, and hybridization of cDNA and cRNA to 4 × 44 K Agilent Barley Gene Expression Arrays (Agilent Technologies) were carried out at the Genomics Core Facility, European Molecular Biology Laboratory (EMBL), Heidelberg, Germany, as described earlier in Kwasniewski et al. (2016). The microarray data were analyzed using GeneSpring GX 12.5 software (Agilent Technologies). Hybridization data were subjected to per chip normalization using the percentile shift method to the 75th percentile. A baseline transformation was then performed to the median of all of the samples. Statistical testing for differential expression was performed using two-way ANOVA followed by the Benjamini–Hochberg false discovery rate (FDR) correction for multiple testing (Benjamini and Hochberg, 1995). Fold change (FC) ≥ 3 (*P* ≤ 0.05 after FDR correction) was considered as differential expression of a gene between drought-treated and control samples. Gene expression changes were also compared between genotypes in control conditions, in order to find genes that may have an impact on a faster drought response of the tolerant genotype. Here, a similar two-way ANOVA analysis was performed, but the stringency was lowered to FC ≥ 2 (*P* ≤ 0.05 after FDR correction), with the assumption that even smaller initial expression differences may be beneficial for the faster drought acclimation and stress response. Raw microarray data, normalized intensity values, and corresponding metadata are accessible through the Gene Expression Omnibus (GEO) repository under accession number GSE103278.

### Agilent barley gene expression microarray annotation and GO enrichment analysis

The sequences of probes from the Agilent Barley Gene Expression Microarray were mapped to cDNAs representing high-confidence (HC) barley genes, based on the barley genome assembly, version 082214v1, available in Ensembl Plants database (http://plants.ensembl.org). PLAZA Monocots (http://bioinformatics.psb.ugent.be/plaza/versions/plaza_v3_monocots; Proost et al., 2015) annotation data were used to annotate mapped sequences. Gene Ontology enrichment analysis was carried out using Singular Enrichment Analysis (SEA) available through AgriGO Toolkit (http://bioinfo.cau.edu.cn/agriGO/analysis.php). Customized analysis was performed using hypergeometric statistical test, at significance level of *p* = 0.01 and minimum mapping entries of 5. The GO data for the whole set of barley genes were retrieved from Ensembl Plants database and used as a background reference for the analysis. Venn diagram was drawn using Venny 2.1 (Oliveros, 2015). The Euclidean distance metric and Ward's linkage method were applied in the hierarchical clustering analysis.

### Quantitative reverse transcription (RT)-qPCR

One microgram of total RNA was subjected to DNase treatment and subsequent cDNA synthesis using RevertAid First Strand cDNA Synthesis Kit (Thermo Scientific) according to the manufacturer's instructions. The cDNA was diluted 1:5 with ddH_2_O and used as a template for the qPCR. The primers that were used in the qPCR were designed using Quant-Prime software (http://www.quantprime.de). The 10 μl qPCR reaction contained 2 μl of cDNA, 1 μl of the primer pair mixture (5 μM), and 5 μl of 2 × Master Mix (LightCycler 480 SYBR Green I Master; Roche). The qPCR protocol for the amplification on LightCycler 480 Real-Time PCR Instrument (Roche) using the SYBR Green I method was as follows: initial denaturation for 10 min at 95°C, followed by 45 cycles of 10 s at 95°C, 15 s at Ta for each primer pair (Supplementary Tables 1, 2), and 10 s at 72°C, followed by a melting-curve analysis. The gene for ADP-ribosylation factor 1 was used as a reference (Rapacz et al., 2012). All analysis were done in three biological replicates. Amplification efficiencies were calculated using LinRegPCR (Ramakers et al., 2003). Calculations of the fold change of expression (FC) were made using Pfaffl method (Pfaffl, 2001). Statistical significance of expression differences between samples were tested using REST software (Pfaffl et al., 2002). Spearman's rank correlation coefficient was used to statistically compare microarray and qPCR FC data. To compare the expression level of selected genes between control samples of both genotypes, the relative expression was shown as a value of (40–dCT); were dCt was the difference between Ct value of a gene of study and a reference gene.

## Results

### A general description of transcriptome changes under drought stress in two genotypes of barley

Gene expression analysis of two barley genotypes subjected to 10-days of drought showed substantial changes of their transcriptomes both in leaves and roots. From around 1600 to 3500 of microarray probes showed differences in hybridization signals between samples from drought-treated and control conditions (Supplementary Data 1–4). After annotation of microarray probes with the use of information of high confidence (HC) genes of barley, available in PLAZA Monocots database, these numbers translated to more than 600–1,600 differentially expressed genes (DEGs; Table 1). Drought stress resulted in stronger transcriptional changes in Maresi variety than in CamB genotype in comparison to optimal growth conditions. In the same time, a similar number of DEGs were found in leaves and roots within each genotype. Also, a comparable number of DEGs were either down- or up-regulated upon drought stress within a genotype.

**Table 1:** Summary of differentially expressed genes in barley genotypes CamB and Maresi under drought stress in comparison to optimal water supply (control conditions).

**Genotype**	**Organ**	**No. of probes**		No. of genes with known annotations^*^	
		**Down-regulation**	**Up-regulation**	**Down-regulation**	**Up-regulation**
CamB	Leaf	1,589	2,811	727	1,003
CamB	Roots	2,453	1,628	1,118	662
Maresi	Leaf	3,540	3,639	1,556	1,441
Maresi	Roots	3,720	3,736	1,670	1,555

*P < 0.05; fold change (FC) ≥ 3*.

* *Barley high confidence genes*.

**Figure 1:**
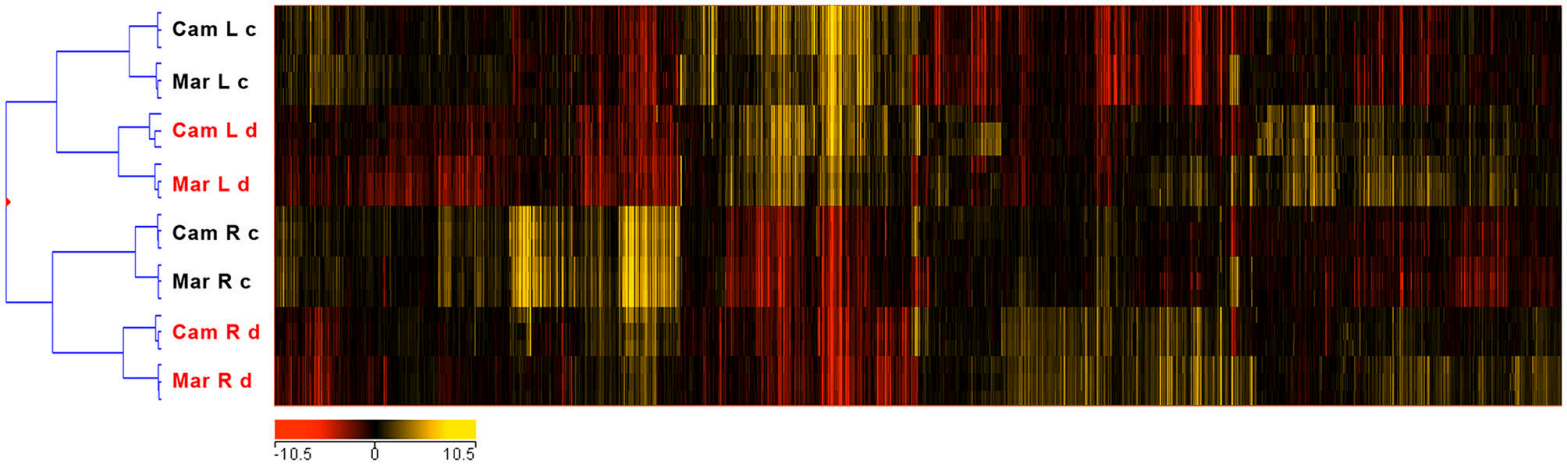
Hierarchical clustering of microarray data. Cam, CamB genotype; Mar, Maresi genotype; L, leaves; R, roots; c, control conditions; d, drought stress.

Hierarchical clustering of samples showed that the most important factor dividing all samples into two major groups is the type of organ indicating that roots and leaves have a distinct pool of actively transcribed genes. The second level of samples clustering was dependent on growth conditions (drought stress or control). The least discriminative factor was the genotype (Figure 1).

**Figure 2:**
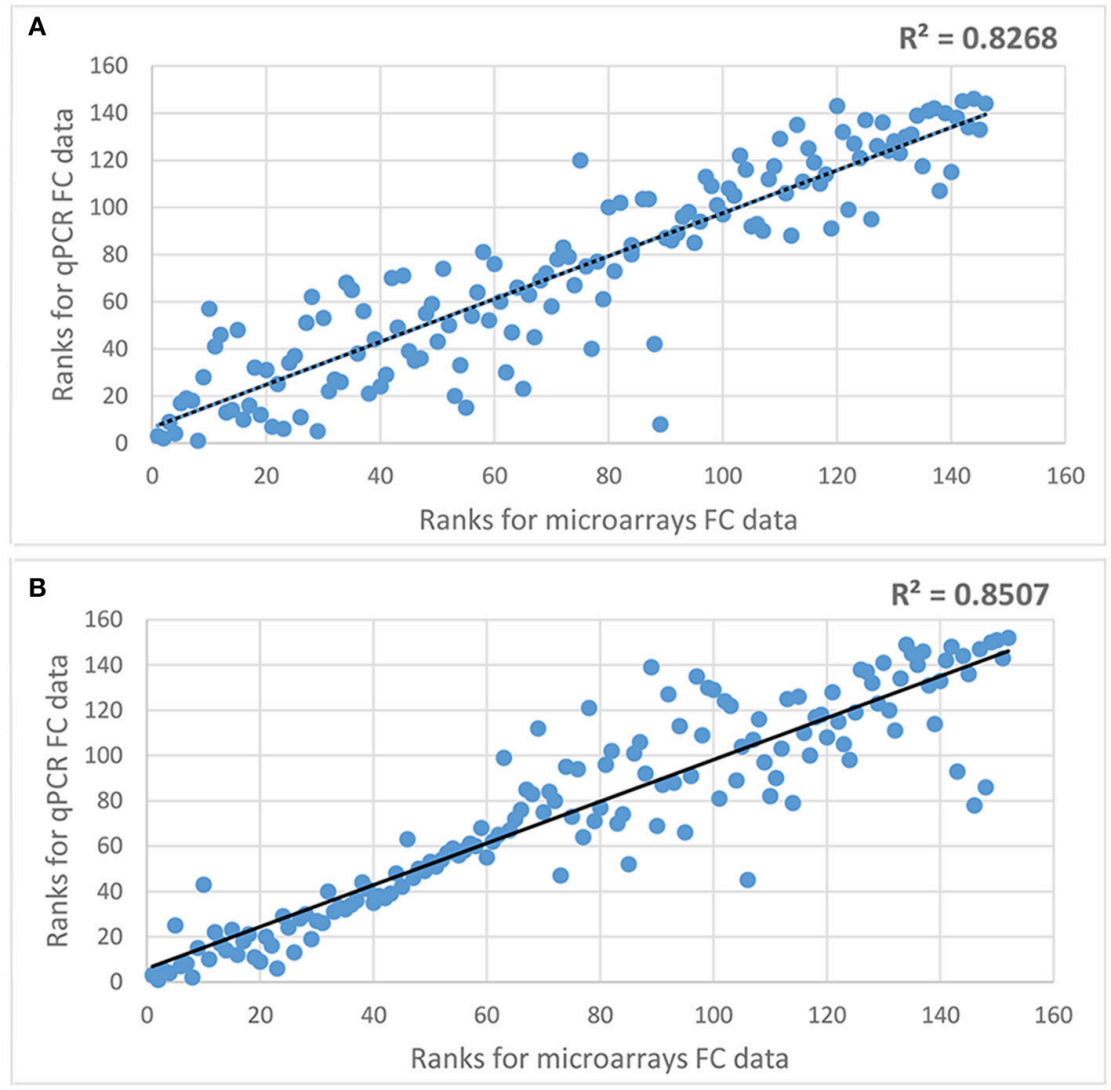
Spearman's rank correlation coefficient analysis comparing microarray and qPCR fold change data for selected DEGs. **(A)** comparison of microarray and qPCR data for genes differentially expressed in roots, **(B)** comparison of microarray and qPCR data for genes differentially expressed in leaves.

In order to check the reliability of microarray data a subset of DEGs were analyzed using qPCR method. It included 73 genes differentially expressed in roots and 76 genes differentially expressed in leaves of either both or in one genotype (Supplementary Tables 1, 2). Spearman's rank correlation coefficient analysis showed a very high correlation in a fold change of expression in both types of analysis (correlation coefficient of 0.8268 and 0.8507 for data from roots and leaves, respectively; Figure 2), what indicates a very high quality of microarray analysis.

**Figure 3:**
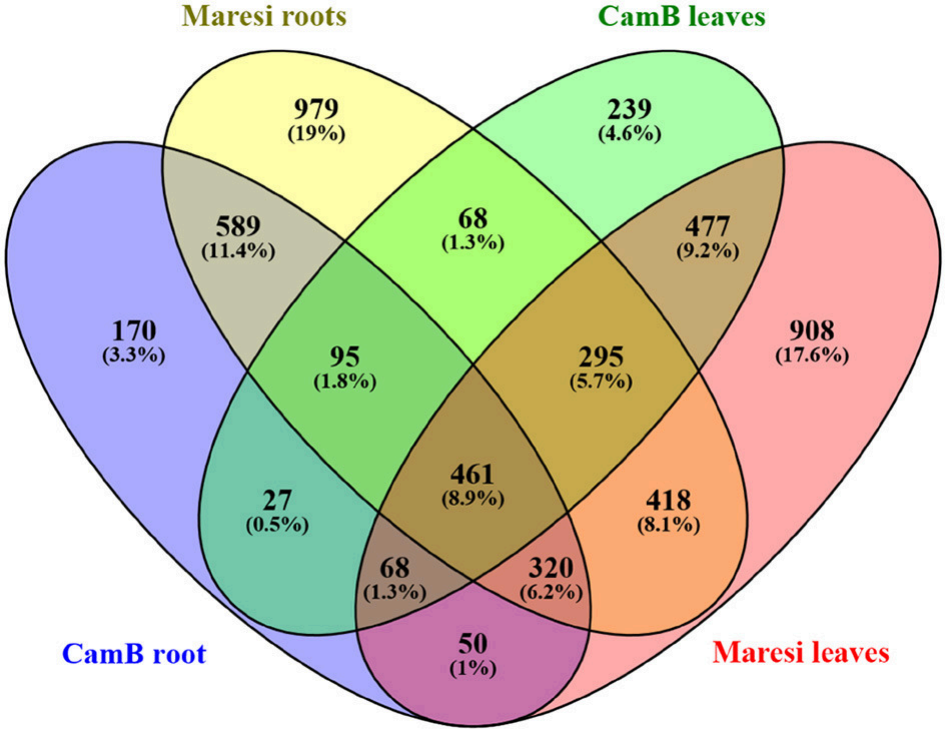
The comparison of a number of differentially expressed genes after drought treatment in CamB and Maresi leaves and roots.

The comparison of drought-influenced DEGs in Maresi and CamB showed that 1802 DEGs were found simultaneously in leaves and roots, although their differential expression varied in both genotypes. Within this group of genes, 461 DEGs were genotype- and organ-independent (Figure 3). GO enrichment analysis showed that the majority of genes with differential expression in both organs belong to the category of small molecule metabolism (Table 2). They include genes involved mostly in amino acid, nucleobase and nucleotide metabolic processes and also the metabolism of other compounds, such as sugars, fatty acids or ketones and have the activity of kinases, phosphatases, methyltransferases, mutases, or lipoxygenases (Supplementary Table 3). Another highly represented category of processes was the establishment of localization, where many transporters, channels and carrier proteins related mostly to sugar, carboxylic acid, ions, protons, water and hormone transport were grouped. Genes encoding factors driving the vesicle transport and compound localization into the cell organelle were also found within this category. A high number of genes represented the oxidation reduction processes and other metabolic processes, such as carbohydrate or nitrogen compound metabolism and a generation of precursor metabolites and energy. Drought stress resulted also in differential expression of factors important for cell wall biogenesis and organization. They include proteins involved in the biosynthesis and deposition of cell wall compounds, mainly cellulose and xyloglucan, proteins classified as pathogen-related factors, expansins and transcription factors. Another highly represented category was related to the control of microtubule-based process. Here, down-regulation of genes encoding tubulin, dynein, kinesins and microtubule-associated proteins was observed. Several DEGs related to chromatin remodeling encoded mostly H1, H2A, and H2B histones and were also downregulated by the drought stress. Other biological processes involved in general drought response were represented by genes responsible for regulation of signal transduction and hormone-mediated signaling pathway, xylem development, defense response to fungus and response to heat. Interestingly, a group of genes with ontology to photosynthesis process exhibited differential expression upon drought in both roots and leaves. This group consisted of various enzymes and factors with chloroplastic localization: factors regulating RNA processing and translation in chloroplasts, photosystem I and II compounds, enzymes important for the synthesis of electron transport cofactors and redox reactions (Table 2, Supplementary Table 3).

**Table 2:** List of significantly enriched gene ontologies representing biological processes related to organ-independent drought response.

**GO term**	**Description**	**No. of genes in the input list**	**No. of genes in the reference list**	***p*-value**
**METABOLISM**				
GO:0005975	Carbohydrate metabolic process	145	1,192	1.9e−05
GO:0034641	Cellular nitrogen compound metabolic process	88	715	0.00041
GO:0006091	Generation of precursor metabolites and energy	47	375	0.0053
GO:0008299	Isoprenoid biosynthetic process	10	231	0.023
GO:0044281	Small molecule metabolic process	214	2,110	0.0065
**CELL WALL**				
GO:0042546	Cell wall biogenesis	22	121	0.00054
GO:0044036	Cell wall macromolecule metabolic process	20	124	0.0041
GO:0071555	Cell wall organization	28	206	0.0094
GO:0030243	Cellulose metabolic process	16	84	0.0018
**TRANSPORT**				
GO:0051234	Establishment of localization	197	1,951	0.0098
GO:0007017	Microtubule-based process	8	182	0.036
**SIGNALING**				
GO:0006333	Chromatin assembly or disassembly	5	76	0.02
GO:0009755	Hormone-mediated signaling pathway	26	139	0.00011
GO:0009966	Regulation of signal transduction	21	142	0.009
**STRESS RESPONSE**				
GO:0050832	Defense response to fungus	13	71	0.0064
GO:0009408	Response to heat	6	97	0.015
Development:				
GO:0010089	xylem development	5	15	0.0064
**OTHER**				
GO:0010466	Negative regulation of peptidase activity	7	19	0.00064
GO:0055114	Oxidation reduction	148	1,349	0.0014
GO:0015979	Photosynthesis	33	251	0.0085

### Genes differentially expressed under drought stress exclusively in roots

**Figure 4:**
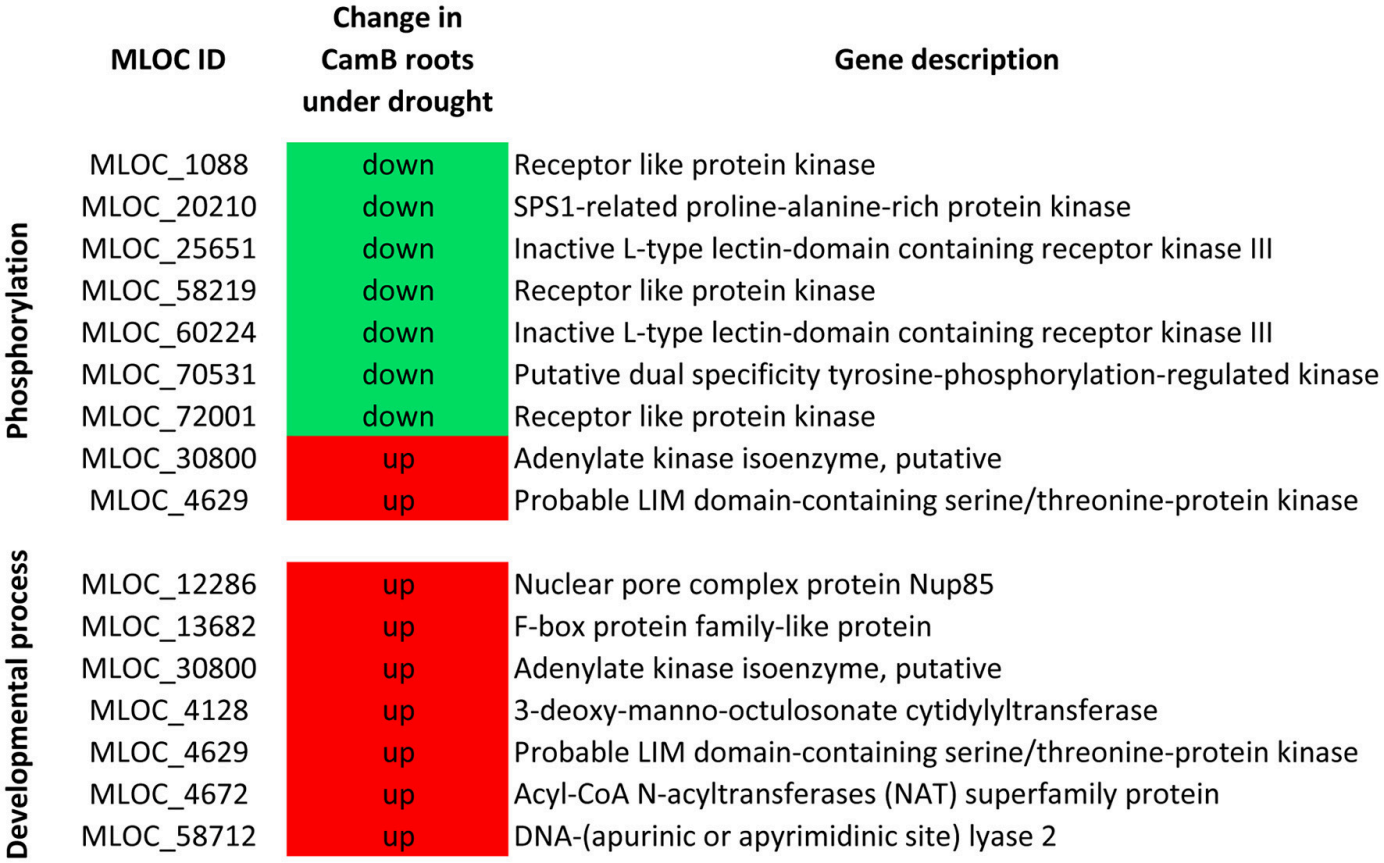
Biological processes significantly enriched after GO analysis of 170 genes differentially expressed exclusively in CamB roots.

From all genes with expression changes under drought condition, 1738 were differentially expressed exclusively in roots. Within this group 170 DEGs were found in CamB roots only, 979 DEGs were characteristic to Maresi roots and 589 showed differential expression in roots of both genotypes. The biological processes of the highest significance after GO enrichment analysis of these 1738 genes included cell division, regulation of DNA metabolism and replication. The highest number of genes were grouped into small molecule metabolism (222 transcripts), lipid metabolism (100 transcripts), signal transduction (62 transcripts) and cell cycle (47 transcripts). Other significantly enriched processes included various aspects of cell metabolism. Five DEGs had ontology to fructose metabolic process and most of them were up-regulated in Maresi roots only. Seven genes that belonged to lignin metabolic process were down-regulated and their differential expression was characteristic for both genotypes. Another seven genes showed ontology with myo-inositol hexakisphosphate biosynthetic process and most of them were up-regulated in roots of Maresi cultivar. We had also found a number of genes from ATP metabolism, brassinosteroid mediated signaling, regulation of response to stress, microtubule-based movement, proteasomal protein catabolism or some aspects of DNA metabolism (Table 3, Supplementary Table 4). Several biological processes including brassinosteroid mediated signaling, fructose metabolism, nucleolus organization and myo-inositol hexakisphosphate biosynthesis contained DEGs that were found in Maresi roots only. There was no opposite category–no genes of significant GOs were found to be specific only to CamB roots, when the GO analysis was done for all 1738 genes simultaneously. For this reason, the list of 170 DEGs, which were differentially expressed only in CamB roots were analyzed separately in terms of their gene ontology. Two significantly enriched categories of biological processes emerged: phosphorylation process, with seven down-regulated and two up-regulated genes encoding several protein kinases and the developmental process, where seven up-regulated genes were found (Figure 4).

**Table 3:** List of significantly enriched gene ontologies representing biological processes involved in drought response exclusively in roots.

**GO term**	**Description**	**No. of genes in the input list**	**No. of genes in the reference list**	***p*-value**	**Number of DEGs in:**		
					**CamB only**	**Mar only**	**CamB and Mar**
**METABOLISM**							
GO:0046034	ATP metabolic process	24	174	0.0091	4	11	9
GO:0006000	Fructose metabolic process	5	16	0.0076	0	4	1
GO:0009808	Lignin metabolic process	7	26	0.0041	0	1	6
GO:0006629	Lipid metabolic process	100	918	0.0034	7	56	37
GO:0010264	Myo-inositol hexakisphosphate biosynthetic process	7	30	0.0096	0	6	1
GO:0010498	Proteasomal protein catabolic process	11	58	0.0071	3	4	4
GO:0051052	Regulation of DNA metabolic process	22	99	1.60E−05	1	16	5
GO:0044281	Small molecule metabolic process	222	2110	0.00027	23	129	70
**CELL DIVISION**							
GO:0007049	Cell cycle	47	372	0.0025	5	21	21
GO:0051301	Cell division	30	168	5.00E−05	3	14	13
GO:0008283	Cell proliferation	18	95	0.00071	1	12	5
GO:0006261	DNA-dependent DNA replication	23	124	0.0002	1	13	9
**TRANSPORT**							
GO:0007018	Microtubule-based movement	14	59	0.00025	0	9	5
**SIGNALING**							
GO:0009742	Brassinosteroid mediated signaling pathway	5	14	0.004	0	4	1
GO:0007165	Signal transduction	62	551	0.0088	6	36	20
**STRESS RESPONSE**							
GO:0080134	Regulation of response to stress	17	108	0.0072	0	11	6
**OTHER**							
GO:0007000	Nucleolus organization	5	9	0.00036	0	4	1
				IN TOTAL	54	351	214

Around 60 from 1738 genes were grouped into a signal transduction process, which includes important mechanisms of environmental sensing. They represent members of receptor-like kinases, kinases interacting with calcineurin B-like protein (CBL), members of LIM domain serine/threonine-protein kinases or protein phosphatases, MAPK kinases and several members of small GTPases (Table 4, Supplementary Table 4). They were found as DEGs in both genotypes with either up- or down-regulation. Similarly, some genes related to ATP metabolism with sequence similarity to various members of ABC transporter family may be important for sensing mechanisms operating in roots (Table 5, Supplementary Table 4).

**Table 4:** Selected genes from signal transduction processes, which ontologies did not overlap with other biological processes, involved in drought response in roots of CamB and/or Maresi genotypes.

**Gene ID**	**Direction of gene expression changes**		**Functional description**
	**CamB roots**	**Mar roots**	
MLOC_56183		up	Calcium-binding protein
MLOC_66148	down		Calcium-dependent membrane-binding proteins
MLOC_1615		up	Ethylene receptor
MLOC_12021	down	down	Kinase interacting with CBL
MLOC_38536	up	up	Kinase interacting with CBL
MLOC_37916		up	Kinase interacting with CBL
MLOC_74559		up	Kinase interacting with CBL
MLOC_52084		down	LIM domain serine/threonine-protein kinase
MLOC_34990	up	up	LIM domain serine/threonine-protein kinase
MLOC_27126		up	LIM domain serine/threonine-protein kinase
MLOC_4629	up		LIM domain serine/threonine-protein kinase
MLOC_4609		down	MAPK kinase
MLOC_64743		down	MAPK kinase
MLOC_11225		up	Phosphoinositide phospholipase C (PLC)
MLOC_11730		down	Polyamine transporter
MLOC_68242	down	down	Protein kinase
MLOC_51888	down	down	Protein phosphatase
MLOC_36395		up	Protein phosphatase
MLOC_63900		up	Protein phosphatase
MLOC_37049		down	Guanine nucleotide exchange protein
MLOC_36731		down	Rac-like GTP-binding protein, small GTPase
MLOC_18432		down	Ras-related protein Rab, small GTPase
MLOC_61634		down	Ras-related protein Rab, small GTPase
MLOC_73105		down	Ras-related protein Rab, small GTPase
MLOC_5009	down	down	Rho GTPase-activating protein, small GTPase
MLOC_64799	down	down	Rho GTPase-activating protein, small GTPase
MLOC_14604	down	down	Receptor-like protein kinase
MLOC_56855	down	down	Receptor-like protein kinase
MLOC_68443	down	down	Receptor-like protein kinase
MLOC_11190		down	Receptor-like protein kinase
MLOC_57599		down	Receptor-like protein kinase
MLOC_63199	up	up	Receptor-like protein kinase
MLOC_55487	down		Receptor-like protein kinase
MLOC_58219	down		Receptor-like protein kinase
MLOC_63678	down	down	Receptor-like protein kinase
MLOC_3955		down	Transcriptional activator APRR4

**Table 5:** Genes homologous to the ABC transporter family involved in drought response in roots of CamB and Maresi genotypes.

**Gene ID**	**Direction of gene expression changes**		ABC transporter family^*^
	**CamB roots**	**Mar roots**	
MLOC_5108	down	down	ABC transporter B family member 4 (ABCB4)
MLOC_57925	down	down	ABC transporter B family member 4 (ABCB4)
MLOC_58493	down	down	ABC transporter A family
MLOC_66404	down	down	ABC transporter G family
MLOC_51103		down	ABC transporter D family member 1 (ABCD1)
MLOC_62631	up	up	ABC transporter G family member 11 (ABCG11)
MLOC_76366	up	up	ABC transporter C family member 10 (ABCC10)
MLOC_77691	up	up	ABC transporter C family member 15 (ABCC15)
MLOC_43902		up	ABC transporter G family member 37 (ABCG37)
MLOC_55872		up	ABC transporter A family member 7 (ABCA7)
MLOC_242	down		ABC transporter B family member 25 (ABCB25)
MLOC_55616	down		ABC transporter I family member 19 (ABCI19)
MLOC_56945	up		ABC transporter F family member 3 (ABCF3)
MLOC_59318	up		ABC transporter G family member 22 (ABCG22)

* *Description based on sequence similarity to genes from other monocot species*.

### Genes differentially expressed under drought stress exclusively in leaves

Exposition of CamB and Maresi genotypes to drought stress resulted in the differential expression of 1628 genes exclusively in leaves. Similarly to the root transcriptome response, the lowest number of DEGs, 239, were found in CamB genotype only, more genes, 477, showed expression changes in leaves of both genotypes and 908 genes were differentially expressed in leaves of Maresi genotype only. Gene ontology enrichment of these 1628 genes showed that they belong to several processes related to pigment metabolism, including chlorophyll metabolic processes, to the response to light, energy generation, oxygen and reactive oxygen species (ROS) metabolism, transport (ion or monovalent inorganic cation transport, mitochondrial transport or protein localization in organelle), reproduction, morphogenesis and organelle organization, RNA metabolism, and other metabolic processes, such as carboxylic acid metabolism, secondary metabolic process or lipid and small molecule metabolic processes. The last two processes were also found to be significantly enriched in roots, but they include different DEGs than in leaves (Table 6, Supplementary Table 5).

**Table 6:** List of significantly enriched gene ontologies representing biological processes involved in drought response exclusively in leaves.

**GO term**	**Description**	**No. of genes in the input list**	**No. of genes in the reference list**	***p*-value**	**Number of DEGs in**		
					**CamB only**	**Mar only**	**CamB and Mar**
**METABOLISM**							
GO:0046394	Carboxylic acid biosynthetic process	61	454	1.70E−05	4	34	23
GO:0006725	Cellular aromatic compound metabolic process	42	312	0.00028	3	22	17
GO:0034641	Cellular nitrogen compound metabolic process	97	715	6.30E−08	16	48	33
GO:0015994	Chlorophyll metabolic process	23	101	1.90E−06	4	11	8
GO:0051186	Cofactor metabolic process	62	439	3.10E−06	10	31	21
GO:0006091	Generation of precursor metabolites and energy	44	375	0.0033	9	25	10
GO:0006629	Lipid metabolic process	98	918	0.00073	10	55	33
GO:0006740	NADPH regeneration	18	118	0.0037	2	12	4
GO:0006730	One-carbon metabolic process	45	400	0.0065	9	31	5
GO:0042440	Pigment metabolic process	40	179	7.80E−10	8	16	16
GO:0019748	Secondary metabolic process	49	365	0.0001	6	28	15
GO:0044281	Small molecule metabolic process	231	2110	1.80E−07	34	137	60
**RNA RELATED-PROCESSES**							
GO:0034660	NcRNA metabolic process	37	315	0.0064	10	19	8
GO:0009451	RNA modification	32	178	5.50E−06	9	18	5
**TRANSPORT**							
GO:0034220	Ion transmembrane transport	13	70	0.0023	2	7	4
GO:0006839	Mitochondrial transport	19	76	3.30E−06	2	15	2
GO:0015672	Monovalent inorganic cation transport	22	141	0.0011	2	15	5
GO:0033365	Protein localization in organelle	21	128	0.00072	1	18	2
**STRESS RESPONSE**							
GO:0010218	Response to far red light	7	29	0.0052	0	4	3
GO:0009744	Response to sucrose stimulus	10	53	0.0062	0	6	4
**OTHER**							
GO:0006996	Organelle organization	107	1022	0.00085	14	70	23
GO:0006800	Oxygen and reactive oxygen species metabolic process	14	85	0.0049	1	8	5
GO:0009886	Post-embryonic morphogenesis	20	139	0.0046	2	13	5
GO:0048610	Reproductive cellular process	14	92	0.0099	0	13	1
				IN TOTAL:	178	747	364

The most important role of leaves is to conduct carbon assimilation and drought stress strongly influences the processes important for photosynthesis, chloroplast function and energy generation. Our study shows that the expression of several genes involved in pigment metabolism, chlorophyll metabolic processes and response to light is influenced by this stress. They include genes encoding light harvesting proteins, electron transport chain, pigment synthesis, signaling, redox reactions or Calvin cycle, and also genes controlling the process of translation in chloroplasts. Additionally, several genes, out of 1628 analyzed, were involved in the regulation of cytoskeleton organization and were either up- or down-regulated in Maresi leaves (Supplementary Table 5). Organelle organization requires efficient intracellular transport of various compounds. Drought stress resulted in Maresi-specific up-regulation of several genes encoding subunits of vacuolar type proton ATPase, and CamB-specific up-regulation of ATP synthase, ADT/ATP carrier protein and calcium-transporting ATPase. In the same time, genes important for mitochondrial transport were mainly down-regulated in Maresi cultivar (Supplementary Table 5). Similarly to roots, the biggest number of DEGs have fallen into various categories of metabolic processes, including one-carbon metabolic process, nitrogen metabolism or carboxylic acid biosynthesis, but many of them showed also overlapping ontologies between these biological processes (Supplementary Table 5).

### Genes shaping drought tolerance in barley

In order to select genes that may be involved not only in drought response, but in drought tolerance, we have compared the lists of genes that were differentially expressed after drought stress with the genes that showed different level of expression under optimal water supply (control conditions) between CamB and Maresi genotype (Supplementary Data 5, 6). We assumed that CamB genotype, which showed to be more drought tolerant based on physiological analysis, may exhibit a higher level of expression of certain genes under control conditions, while in Maresi these genes start to be up-regulated only after the occurrence of drought. And vice-versa, some sub-set of genes with a lower initial expression in CamB may be down-regulated just after drought stress in Maresi. Such comparisons allow us to select candidates that may play a role in better adaptation to drought in the case of CamB and may explain the generally lower number of DEGs found in this tolerant genotype after the stress treatment. Altogether, 170 of this type of genes were selected as being differentially expressed exclusively in roots, 237 genes showed this characteristics exclusively in leaves and 99 were found in both organs (Supplementary Table 6). Ten candidate genes were additionally examined by qPCR and this analysis confirmed their initially higher or lower expression in either leaves or roots of CamB in comparison to Maresi (Supplementary Figures 1, 2). A subset of the candidate genes was also differentially expressed in CamB after drought treatment, although their fold change between control and drought conditions was usually smaller than in Maresi genotype (Supplementary Data 1–4). From among 170 candidates of initially different expression in roots, 56 were also differentially expressed in this organ under the stress in CamB. Out of 237 genes, a group of 56 transcripts were drought-DEGs in CamB leaves and from among 99 genes characterized by higher or lower initial expression in both CamB organs, 41 and 42 genes were differentially expressed under drought in CamB roots and leaves, respectively (Supplementary Table 6). The candidates that may shape drought tolerance were further compared to different categories of DEGs presented in Figure 3. This analysis showed that they may be found within DEGs detected in leaves and/or roots of Maresi cultivar or within the general drought response genes (Supplementary Figure 3).

**Figure 5:**
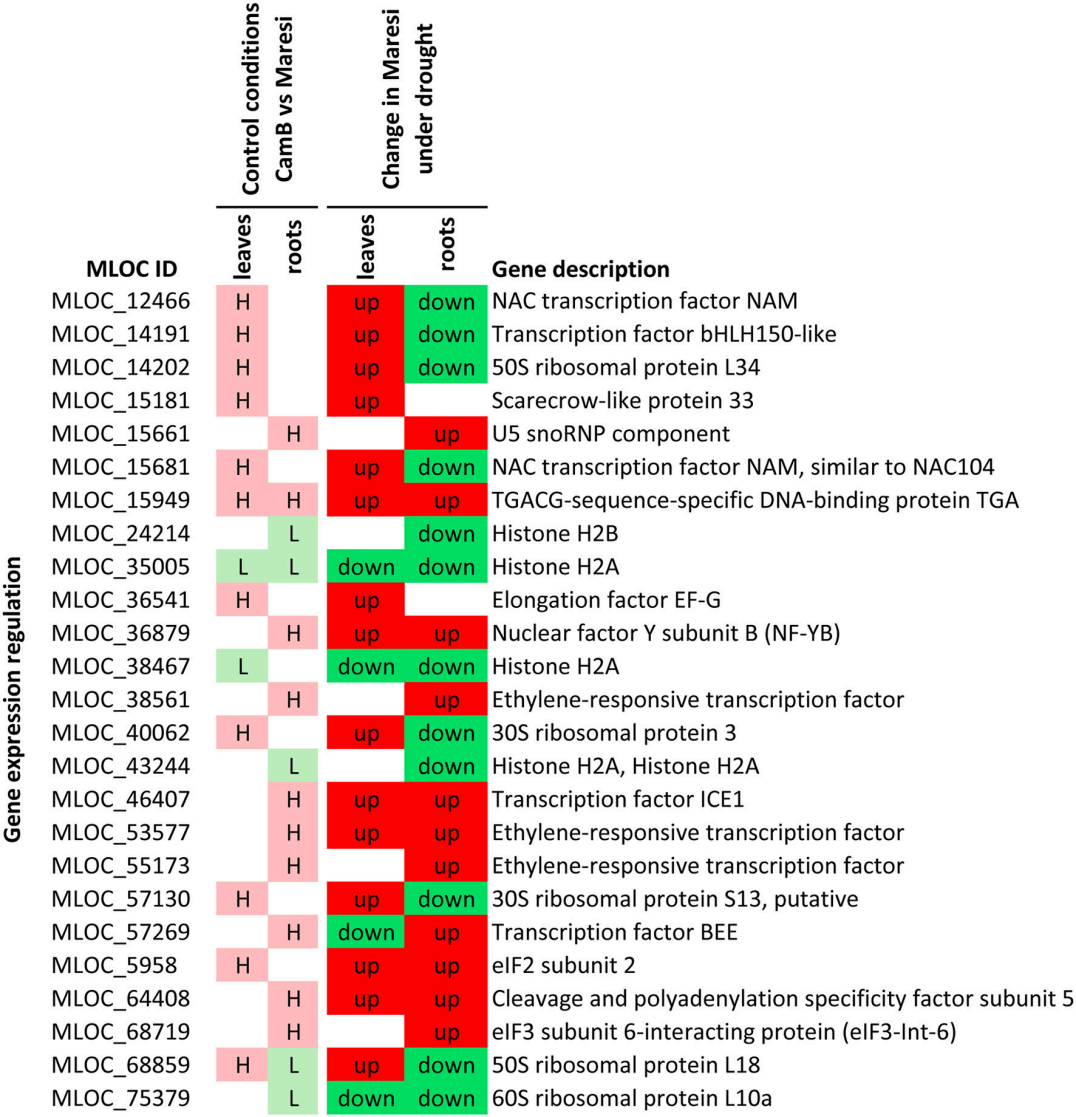
Selected genes putatively involved in drought tolerance in barley, that play a role in gene expression regulation processes. H–genes with higher expression in CamB than in Maresi in control conditions, L–genes with lower expression in CamB than in Maresi in control conditions, up–up-regulation of a gene in Maresi after drought treatment, down–down-regulation of a gene in Maresi after drought treatment. Gene description was based on the annotations available in Plaza Monocots database.

**Figure 6:**
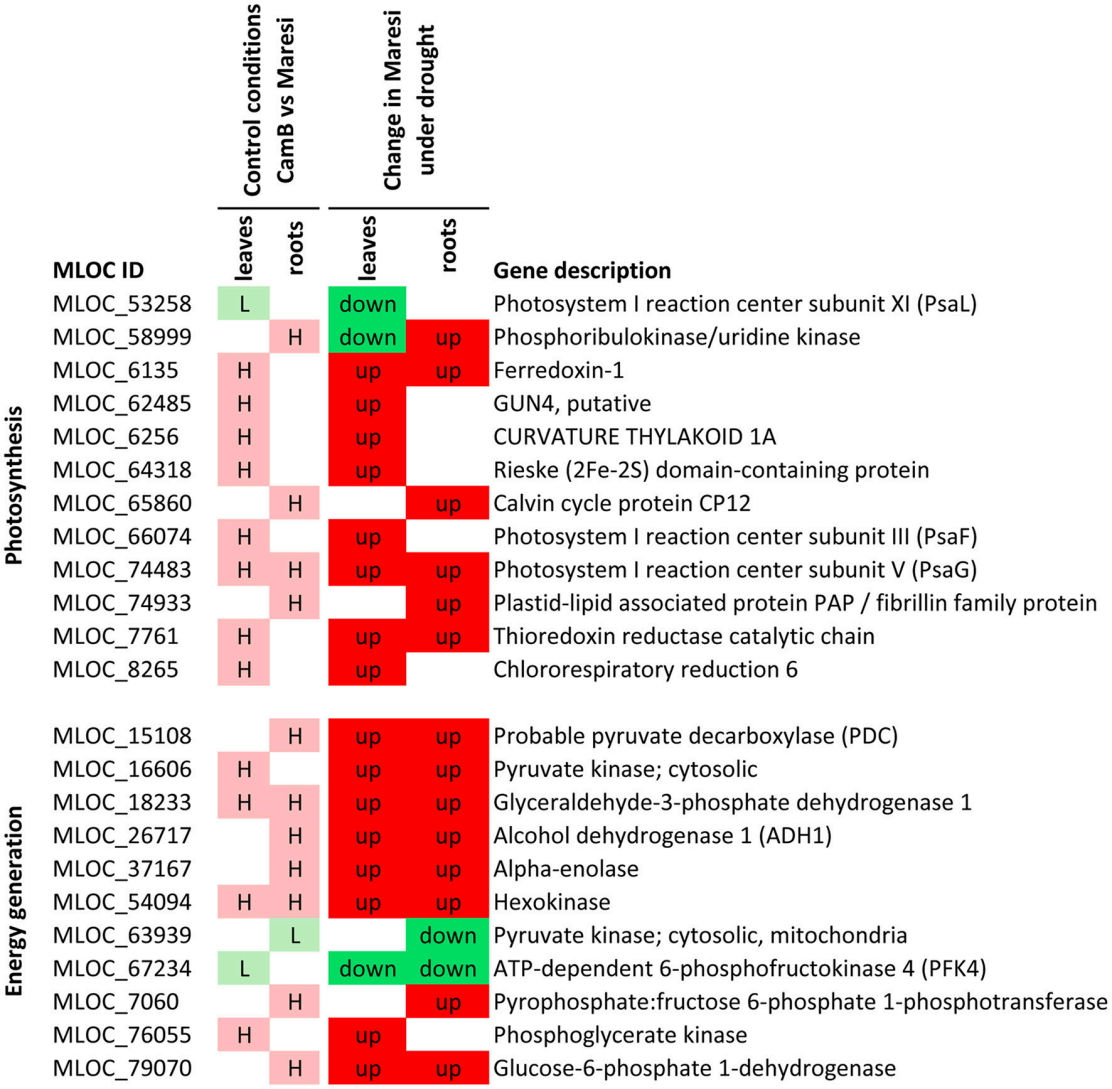
Selected genes putatively involved in drought tolerance in barley, that play a role in photosynthesis and energy regulation processes. H–genes with higher expression in CamB than in Maresi in control conditions, L–genes with lower expression in CamB than in Maresi in control conditions, up–up-regulation of a gene in Maresi after drought treatment, down–down-regulation of a gene in Maresi after drought treatment. Gene description was based on the annotations available in Plaza Monocots database.

**Figure 7:**
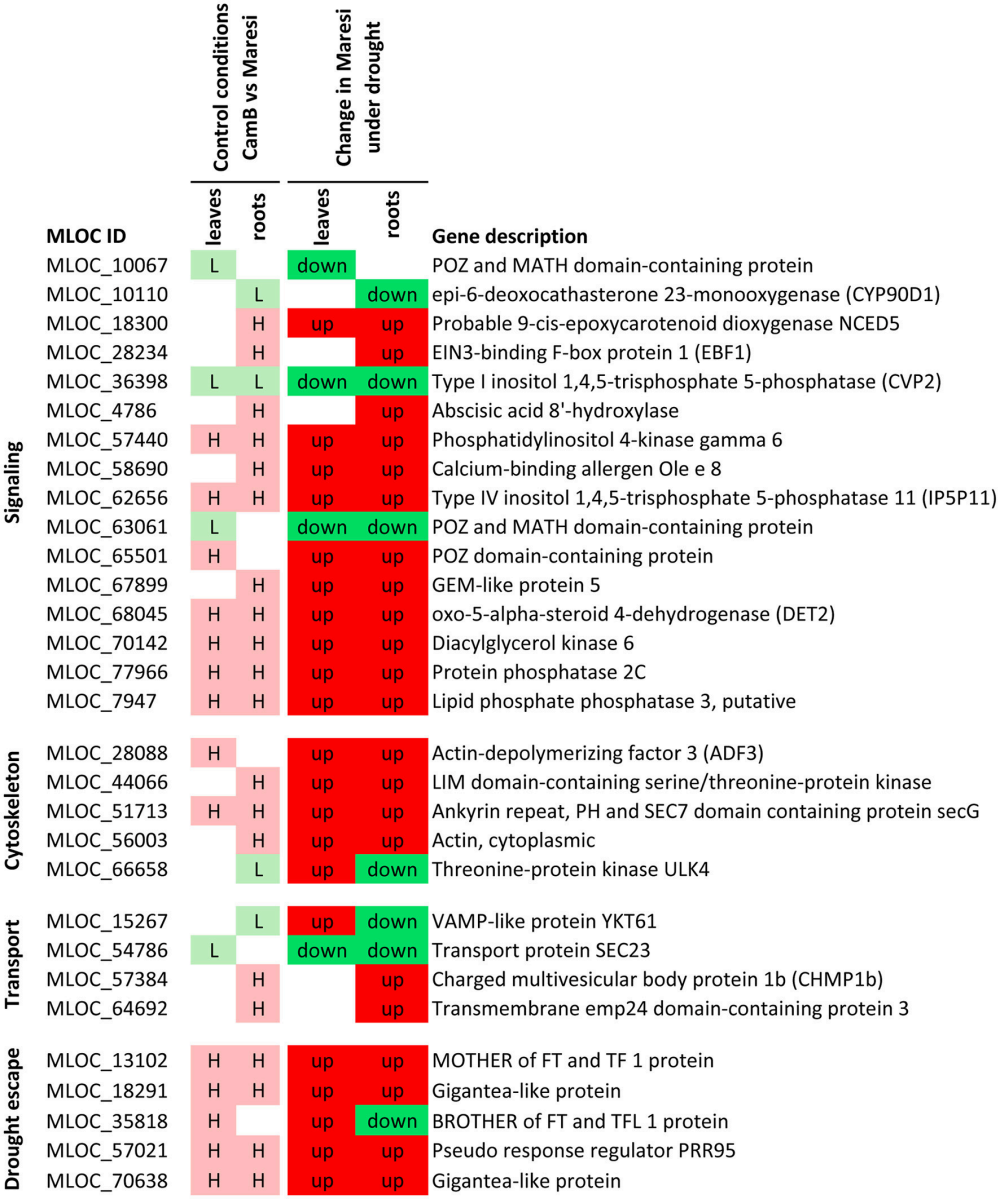
Selected genes putatively involved in drought tolerance in barley, that play a role in signal transduction, cytoskeleton formation, vesicle transport and drought escape processes. H–genes with higher expression in CamB than in Maresi in control conditions, L–genes with lower expression in CamB than in Maresi in control conditions, up–up-regulation of a gene in Maresi after drought treatment, down–down-regulation of a gene in Maresi after drought treatment. Gene description was based on the annotations available in Plaza Monocots database.

Based on a joined analysis of gene ontologies, the functional annotation of transcripts and literature data examination, it was possible to group a sub-set of these genes into several functional categories, including gene expression regulation, photosynthesis and energy generation processes, involvement in signaling processes, cytoskeletal formation, cellular transport and drought escape mechanisms (Figures 5–7). These genes are further extensively discussed in the light of possible mechanisms of drought tolerance in barley.

## Discussion

### Changes in roots and leaves transcriptomes under drought stress

Our analysis showed a substantial changes in roots and leaves transcriptomes of barley seedlings after 10 days of severe drought stress. Such response was largely expected, as strong drought stress has a prominent effect on plant metabolism, which has to be adjusted to adverse conditions to enable plant survival. The largest groups of genes differentially expressed in both organs play a role in various aspects of plant metabolism: amino acids, carbohydrates, lipids, nucleotides or cell wall compounds metabolism. Many of them play a role in the defense against oxidative stress, heat or in various aspects of cellular transport. These processes have already been reported by others, as involved in shaping drought-response in plants (Shaar-Moshe et al., 2015). Here, we would like to emphasize the importance of two phenomena, which are less extensively discussed elsewhere.

The most basic level of drought response in both of studied organs relates to chromatin remodeling, gene expression regulation and RNA processing. A significant number of DEGs related to these processes have their localization in plastids and mitochondria. The gene onthology analysis have grouped them into particular organellar processes, but several had their localization in nucleus, nucleolus and cytoplasm. Part of them represent transcription factors that belong to APETALA2/ethylene-responsive factors (AP2/ERF), bHLH, NAC, WRKY, or ERF families, which are associated with drought tolerance (Sahoo et al., 2013; Janiak et al., 2016). Others include histone-encoding genes or RNA polymerases required for nucleosome modifications, such as a gene similar to TAF5-like RNA polymerase II, which in human is a component of PCAF complex involved in histone H3 acetylation (Brown et al., 2000). Another example is a gene encoding RNA polymerase II subunit RPB2 required for mRNA and many non-coding RNAs transcription. Additionally, genes for RNA splicing machinery, ribosomal proteins and translation initiation and elongation factors were among root and/or leaves DEGs, what reflects the necessity to rebuild a whole expression regulation system in order to respond to drought stress. Many of these DEGs are usually considered as housekeeping genes with relatively stable expression pattern. Our data suggest that the strong drought stress may change plant transcriptome so extensively, that even the basic metabolism is shifted to a completely different level, compared to optimal water conditions. Importantly, such a shift is characteristic primarily to drought sensitive genotype, indicating that drought tolerance may partially rely on some type of readiness of regulatory mechanisms, which helps to answer to drought stress more quickly. We propose and discuss several specific mechanisms of such tolerance in the last section of the paper.

Another and rather unexpected result was the observation of drought-influenced expression of genes related to photosynthesis process, not only in leaves, but also in roots (Table 7). They showed, however, different expression patterns across the samples, what may reflect differences in drought tolerance of studied genotypes. Several DEGs in this group are required for electron chain assembly and their up-regulation in leaves may be important to secure photosynthesis. This may be true for 1,4-dihydroxy-2-naphthoate phytyltransferase, up-regulated in all samples, which is required for the synthesis of phylloquinone, an early acceptor of electrons in PSI. The study of Chlamydomonas showed that phylloquinone is essential for the maintenance of photosynthesis in high light conditions (Emonds-Alt et al., 2017), so up-regulation of genes responsible for its synthesis in leaves may result in a better drought survival. Similar function may have up-regulation of genes that control an oxygen evolving complex (OEC), ferredoxin and a putative ferredoxin-NADP reductase, although their differential expression in leaves was noticed only in Maresi. Interestingly, down-regulation of transcripts for oxygen-evolving enhancer protein 1 (OEE1), PsbQ-like protein 3 and chlorophyll b binding protein 1B-20 was observed in leaves of Maresi and in roots of both genotypes. The up-regulation of ferredoxin-encoding gene was also found in Maresi roots. Such observation may be a result of an adverse effect of drought on a susceptible cultivar, although the answer may also be more complex. We may assume that changes in leaf chloroplasts in susceptible genotype under drought stress are more dynamic and down-regulation of genes essential for photosystem functioning reflects a temporal imbalance between up- and down-regulated genes, when chloroplast metabolism is being adjusted to adverse conditions. Importantly, samples collected in our study represent only one time-point–the end of 10-days stress, so we cannot exclude the existence of fluctuations of expression for a subset of observed DEGs over time.

**Table 7:** Selected DEGs involved in photosynthesis detected in leaves and roots of at least one barley genotype.

**Gene ID**	**Direction of gene expression changes**				Gene description^*^
	**CamB leaves**	**Mar leaves**	**CamB roots**	**Mar roots**	
MLOC_17228		up		up	Oxygen-evolving enhancer protein 3-1 (PsbQ)
MLOC_23254		up		up	Uncharacterized sugar kinase
MLOC_34548		up	up	up	Oxygen-evolving enhancer protein 3-1 (PsbQ)
MLOC_36344		up		up	Iron-responsive element-binding protein 2
MLOC_36541		up	up		Elongation factor EF-G
MLOC_37738	up	up	up	up	1,4-dihydroxy-2-naphthoate phytyltransferase
MLOC_44346		up		up	Apoptotic serine protease NMA111
MLOC_44755		down	down	down	Chlorophyll b binding protein 1B-20 (Lhc A4)
MLOC_44795		up	up	up	Ribulose bisphosphate carboxylase small chain
MLOC_4813		down	down	down	PsbQ-like protein 3 (PsbQL3)
MLOC_48344	up			down	Thylakoid lumenal 15 kDa protein 1
MLOC_53258		down	down		Photosystem I reaction center subunit XI (PsaJ)
MLOC_55726	up	up		up	Pentatricopeptide repeat-containing protein
MLOC_58855	up	up		down	DnaJ/Hsp40 cysteine-rich domain-containing protein
MLOC_58999	down	down		up	Phosphoribulokinase/uridine kinase
MLOC_59370	down	up		down	Serine hydroxymethyltransferase
MLOC_5958		up	up	up	Eukaryotic translation initiation factor 2 subunit 2
MLOC_61094		up	up	up	RNA polymerase sigma factor
MLOC_61260		up		up	Protein PROTON GRADIENT REGULATION
MLOC_6135		up		up	Ferredoxin-1 (Fd)
MLOC_62113		up		down	Glucose-1-phosphate adenylyltransferase subunit 2
MLOC_70480		up	up		Ferredoxin-NADP reductase, putative (FNR)
MLOC_74483		up	up	up	Photosystem I reaction center subunit V (PsaG)
MLOC_75003	down	down		down	Rhodanese-like domain-containing protein 9
MLOC_75514		up		up	PsbP domain-containing protein 4 (PsbP)
MLOC_78630		down	down	down	Oxygen-evolving enhancer protein 1 (PsbO)
MLOC_82117	up	up	up		Magnesium-chelatase subunit ChlH

* *Description based on the gene annotations from PLAZA Monocots database; abbreviations in parenthesis are based on KEGG database, and correspond with the data included in Supplementary Figures 4, 5*.

Nevertheless, the accelerated expression of plastid genes is sustained by the up-regulation of genes encoding an organellar RNA polymerase sigma factor and translation initiation and elongation factors that was observed in our study, and again these changes were more prominent in Maresi, the less tolerant genotype. Moreover, a gene encoding a pentatricopeptide repeat-containing protein (MLOC_55726), required for splicing of group II introns in chloroplasts and the assembly of PSI and PSII (de Longevialle et al., 2008), was up-regulated also in CamB leaves. This indicates indirectly that in the drought tolerant genotype the expression level of MLOC_55726 target genes is high enough to produce substrates for splicing and subsequent production of required chloroplast proteins under stress. Such observation is in agreement with physiological and morphological data showing a smaller reduction of photosynthetic activity and the existence of a higher number of well-developed chloroplasts in leaves of drought-treated CamB compared to Maresi (Filek et al., 2015, 2016).

The question of what is the function of the above-mentioned genes in roots under drought stress does not have a straightforward answer. The most self-explanatory possibility may be attributed to a possible dual role of electron transfer system proteins, which may also function to prevent oxidative damage. This may suggest a much broader role of plastids, which in roots are usually associated with starch storage and the setup of symbiotic interactions (Lohse et al., 2005). It seems that during drought stress root plastids may start to play a role of “anti-oxidative centers”, protecting root macromolecules from oxidative damage. Several of these DEGs are members of electron transfer systems in the photosynthesis pathway (Supplementary Figures 4, 5), what may predispose them also to the scavenging of free radicals in reactive oxygen species. An interesting study of chloroplasts development and function in *Arabidopsis* roots was presented by Kobayashi et al. (2013), in the context of root-specific overexpression of *Golden2-like* (*GLK*) gene, which triggered root chloroplast development, and enabled the activation of photosynthesis process in this organ. In addition to *GLK*-overexpression lines the authors analyzed also plastids of non-transgenic wild-type roots. Poor formation of thylakoid membrane was noticed in root plastids, but grana stacks were relatively well developed. It was possible to detect the accumulation of PsbO, D1, and LhcA2 proteins, which form the reaction centers of photosystems I (PSI) and II (PSII). The analysis of fluorescence spectra showed, however, that light-harvesting protein complexes were probably weakly or not coupled to PSI and PSII and that there was a higher energy dissipation in root PSII in comparison to leaf chloroplasts. Additionally, there was a higher ratio of Chl b and carotenoids over Chl a in root plastids than in the chloroplasts of wild type leaves. All this data suggest that root plastids form relatively large antenna complexes per reaction center, what is consistent with the enhanced grana formation and a high carotenoids accumulation that is responsible for thermal dissipation of energy (Kobayashi et al., 2013). Thus, the energy dissipation mechanisms that are highly active in root plastids may be responsible also for their anti-oxidative role during drought stress, by the analogy to the photoprotective function of these mechanisms in leaf chloroplasts (Matsubara et al., 2016). The specific function of several DEGs from presented study that may contribute to this process is discussed later.

It is necessary to mention that differential expression of genes related to chloroplast biogenesis and photosynthesis was found in roots of drought stressed or dehydrated plants also in other species, such as rice (Minh-Thu et al., 2013), cotton (Ranjan and Sawant, 2015), or chickpea (Molina et al., 2008) and the authors usually point to their possible anti-oxidative role. Careful examination of literature resources, including Supplementary Materials, is much more striking: in many papers up-regulation of photosynthesis-related genes, mostly encoding light harvesting or OEC proteins, was recorded in roots under drought, but these findings were not discussed (for example in: Cohen et al., 2010; Lorenz et al., 2011; Moumeni et al., 2011, among others). Universality of these observations opens the question of what is the precise role of root plastids in drought response and is it possible to use these mechanisms to improve drought tolerance.

One technical question, that may arise here, is related to the method of material collection for root transcriptome analysis. It is necessary to remove root system from the soil and temporary expose it to light, what may induce expression of light-regulated genes. The work of Minh-Thu et al. (2013) on rice roots subjected to dehydration stress showed, however, that even when their experiment was conducted in the dark, the expression of selected genes was still induced by dehydration, indicating that the stress itself has a significant impact on the expression of photosynthesis-related genes in roots.

### Root-specific transcriptome changes under drought stress

Roots are the first site of drought signal perception and their environmental sensing role was reflected by the expression changes of genes involved in signal transduction process. One of the earliest signal transduction reaction is driven by phosphorelay pathway, which includes MAPK kinases and phospholipases C (PLC) and D (PLD), that form a regulatory network (Mane et al., 2007) and some of its components respond very rapidly to the stress. It has been shown, for example, that MAPK genes expression is induced in roots just within 1.5 h after stress application, and is equally rapidly decreased afterwards (Peng et al., 2006). This down-regulation may be regulated by PLD, which additionally down-regulates genes encoding members of small GTPases (Mane et al., 2007). Our study showed specific to Maresi roots down-regulation of two MAPK kinases genes, several genes from small GTPase family and a gene for a transcriptional regulator APRR4 with Myb-like motif that is involved in phosphorelay signal transduction system (Schaller et al., 2008). Moreover, two genes with annotation to PLDs (MLOC_4380 and MLOC_56293) were up-regulated in leaves and roots of this genotype. These observations show that in the drought-sensitive genotype a PLD-dependent phosphorely pathway is active in roots after 10 days of strong drought stress, contrary to the tolerant genotype, where it was not detected. Previous work on *Arabidopsis* leaves showed that PLD overexpression has dual consequences: induction of drought response to maintain water status in a short-term, but negative impact on drought tolerance, with decreased RWC and increased membrane oxidation and leakage during a long-term stress (Hong et al., 2008). Our data suggest that similar mechanisms may act in barley roots. The root specificity of detected DEGs presumes that different genes may operate in phosphorely pathway in both organs, with possible involvement of APRR4 transcriptional regulator in this process in roots.

Other signaling genes encoding LIM domain serine/threonine-protein kinases were also detected in roots. Four genes with this annotation were found as DEGs in both genotypes, but with different expression pattern. LIM proteins may function as biosensors that mediate communication between the cytosolic and the nuclear compartments and they are thought to create a link between actin cytoskeleton and transcriptional machinery (Kadrmas and Beckerle, 2004). Their differential expression in roots under drought shows that they may be a very important component of efficient signaling toward stress response. Interestingly, seven genes encoding various types of protein kinases were down-regulated exclusively in CamB roots and two genes of such function were up-regulated. This observation indicates that the tolerant CamB genotype may have different sensitivity to the drought signals than Maresi cultivar, what influences the overall drought response and may be one of the components of drought tolerance mechanisms observed in Syrian genotype.

Additionally, GO enrichment analysis pointed to a group of genes that were up-regulated exclusively in CamB roots and fell into the category of developmental processes. Their annotation shows that they play a very different molecular functions. One of them (MLOC_12286) is similar to a nuclear pore complex protein Nup85, which in *Arabidopsis* is important to maintain mRNA transport from nucleus to cytoplasm (Parry, 2014). A higher expression of this gene in CamB may predispose this genotype to a more efficient transport of transcripts required to regulate drought response in roots. Another gene (MLOC_4128) that encodes 3-deoxy-manno-octulosonate cytidylyltransferase plays a role in biosynthesis of rhamnogalacturonan II (RG-II), which belongs to pectic polysaccharide of the primary cell wall. A study of *Arabidopsis* shows that a mutation in this gene impairs pollen tube elongation (Kobayashi et al., 2011). As the growth of pollen tubes may be controlled by similar mechanisms as tip growth of root hairs, this gene may be one of the candidates to further study in the context of drought tolerance. The next gene, MLOC_4672, was also up-regulated exclusively in CamB roots. It encodes a protein from acyl-CoA N-acyltransferases (NAT) superfamily, which catalyzes N-acetylation of proteins. Such modification plays a role in the control of protein degradation, protein folding or formation of protein complexes (Aksnes et al., 2016) what may have a broad impact on plant development regulation under drought. Another multifunctional gene up-regulated in CamB roots only (MLOC_58712) encodes a DNA-(apurinic or apyrimidinic site) lyase 2. This enzyme is involved in DNA repair, but may also contribute to gene expression regulation, as it increases the affinity of transcription factors to DNA (Babiychuk et al., 1994). Another interesting group of genes differentially expressed in roots of one or both genotypes belongs to ABC transporter superfamily which gathers proteins necessary for the exchange of various compounds (lipids, hormones, secondary metabolites) across membranes (Hwang et al., 2016). Our study shows that drought stress up-regulates transporters involved in lipid transport, including cutin and wax (ABCG11), which are known to increase drought tolerance (Zhu et al., 2014). Other up-regulated genes include proteins involved in glutathione S-conjugates (ABCC15), what may be important in ROS scavenging, and a ABCG37 transporter that is probably involved in exudation of auxin precursors (Ruzicka et al., 2010) and also phenolic compounds (Ziegler et al., 2017). These compounds may serve to mobilize soil nutrients, or to play a role as signals for soil microbiota that establish interactions with plant roots (Sisó-Terraza et al., 2016). It has been shown that certain groups of microbes can modify stress sensing by the plant and increase its biomass production (Zolla et al., 2013), thus a higher level of ABC transporters capable of exudation of microbe-interacting phytochemicals may be another important factor enhancing drought tolerance. Interestingly, only in CamB genotype the increased expression of a gene similar to ABCG22 was noticed, which functions as an ABA transporter (Ji et al., 2014), indicating that ABA transport and also its metabolism in CamB roots may be on the higher and more dynamic level than in Maresi cultivar, as discussed later.

### Genes shaping drought tolerance in barley

Our study showed that the drought sensitive cultivar Maresi exhibited far more expression changes after drought stress than the tolerant CamB genotype. This observation led to the assumption that CamB may have a “stressed-like” transcriptome that is active already in optimal water conditions and this genotype does not need to initiate expression changes to a such degree as the drought sensitive cultivar, when the stress occurs. So, this hypothesis assumes that CamB is prepared to react to stressful conditions even before it perceives the stress. According to this hypothesis we have selected candidate genes and processes that may be responsible for drought tolerance. We selected genes which showed a different level of expression between CamB and Maresi in control conditions, and simultaneously: (i) they were differentially expressed in Maresi only after application of drought and (ii) they followed the direction of expression pattern of the tolerant genotype CamB. We assumed that such DEGs may be involved in a better adaptation to the unfavorable conditions. For the sake of simplicity, we further refer to this type of genes as having a higher or lower initial expression in drought tolerant CamB genotype.

#### Components of gene expression regulation machinery

Changes in chromatin structure is one of the processes important for this regulation. Several genes encoding H2A and H2B histones were found to be expressed at a lower initial level in CamB genotype compared to Maresi cultivar. Down-regulation of the canonical histone genes may be important for their faster replacement by other variants involved in the regulation of drought response genes. It was suggested that the presence of a histone variant H2A.Z in gene bodies promotes variability in the gene expression pattern (Coleman-Derr and Zilberman, 2012). Moreover, the exchange of the core H2A histone with the H2A.Z variant was necessary for the temperature sensing in *Arabidopsis* (Kumar and Wigge, 2010) and for the maintenance of grain yield in Brachypodium during heat stress (Boden et al., 2013). In addition to the expression regulation, specific histone variants participate in DNA repair (Williamson et al., 2012), what may have a crucial role in the maintenance of genome stability during drought stress. The lack of discovery of other histone variants in our study may be related to the lack of microarray probes for their genes. Nevertheless, we may suppose that down-regulation of the canonical histone genes may be an important mechanism driving their replacement by more specific isoforms, resulting in efficient coping with drought.

Several transcription factors (TFs) showed higher initial expression in CamB genotype and up-regulation in Maresi only upon drought stress. Six of them had such characteristics in roots, four in leaves and one in both organs. Interestingly, four of them had opposite expression pattern in roots and leaves after drought stress in both genotypes. Within the candidate TF genes, with higher initial expression in roots of CamB, was *ICE1* which is a regulator of CBF/DREB1 TFs belonging to the AP/ERF family. It recognizes MYC recognition sites (5′-CANNTG-3′) found in the CBF3/DREB1A promoter. Because the constitutive overexpression of CBF transcription factors in transgenic plants increases plant tolerance to abiotic stresses (Zhang et al., 2013), also the enhanced expression of *ICE1* may promote better plant survival under drought. The affinity of ICE1 to the promoter of CBF3/DREB1A and its initial higher expression in roots of the drought tolerant genotype suggest that it may be involved in the regulation of root system development *via* DREB1A factor. A premise to such conclusion is a study of the overexpression of *Arabidopsis DREB1A* gene in the groundnut, which led to a higher root-to-shoot ratio when the plants were subjected to intermittent and terminal drought stress (Jagana et al., 2012). More direct evidence of ICE1 role in root system growth under osmotic stress emerges from a study of its overexpression in *Arabidopsis* and the observation of a longer main root and a higher number of lateral roots in transgenic plants overexpressing *ICE1* (Xu et al., 2014). Although the enhanced expression of *ICE1* in CamB genotype in optimal conditions was found in roots only, its expression was later up-regulated by drought in roots and in leaves of both genotypes. This observation shows that ICE1 plays a more general role in drought response, but the primary effect of ICE1 may be related to the stimulation of root system growth.

Another transcription factor that is not specific to roots only, but seems to play a role in root system development under drought, is the nucleart factor Y subunit B (NF-YB) TF that exhibited an increased initial expression in CamB roots. TFs from this family are highly conserved and bind to CCAAT motifs in the promoter regions of various genes (Ballif et al., 2011). Overexpression of *ZmNFYB2* in maize resulted in a higher chlorophyll content, better stomatal conductance and maintenance of photosynthesis under drought stress (Nelson et al., 2007). Other study, with overexpression of *NFYB2* gene in *Arabidopsis* showed that roots of transgenic plants elongated faster and the expression of a transgene was localized in the tip region of the root (Ballif et al., 2011).

An interesting gene detected in our study, characterized by higher initial expression in roots of CamB, up-regulation of expression in roots of Maresi under the stress, but down-regulation in leaves of both genotypes under drought, encodes BEE transcription factor from bHLH TF family. A literature data suggest that BEE TF plays a role as a positive regulator of brassinosteroids (BR) signaling pathway and is placed in the middle of an antagonistic interaction between BR and ABA response. It was proposed that *BEE* TFs are early response genes induced by BRs through the BRI1 receptor complex and their expression is repressed by ABA through an unknown ABA receptor (Friedrichsen et al., 2002). In *Arabidopsis*, three *BEE* genes were identified and were found to redundantly promote cell elongation (Friedrichsen et al., 2002). These data and our study suggest, that *BEE* TF may act as a promoting factor for root elongation under drought stress, but inhibition of its expression in leaves is related to ABA-mediated drought response, which regulates stomatal closure and triggers the expression of a large number of drought-responsive genes.

An opposite expression regulation under drought stress was also noticed for other TF genes in our study, including two TFs from NAC family. Their expression was initially higher in leaves of CamB and was up-regulated in Maresi leaves after drought stress, but was down-regulated in roots of both genotypes. NACs belong to a large family of TFs and were found to be differentially expressed under a variety of abiotic stresses, often showing opposite expression changes depending on the type of stress and/or plant organ (Cohen et al., 2010; Huang et al., 2016). It is difficult to predict, what is the precise function of NAC genes detected in our study in shaping plant tolerance to drought. One of them, however, shows a high similarity to *NAC104* (*XND1*) gene, which in *Arabidopsis* was found to be involved in xylem development by a negative regulation of secondary cell wall fiber synthesis (Zhao et al., 2008). Overexpression of this gene led to the suppression of xylem development and plant dwarfism (Zhao et al., 2008). We may speculate that the drought-stimulated down-regulation of *NAC104* gene in roots detected in our study is related to root growth maintenance and vascular tissue development that is necessary for water uptake.

Regulation of drought responsive genes frequently relies on a cascade of action of several transcription factors. An example of such relationship is the existence of a motif of 5′-TGACG-3′ sequence that is found in the promoter of many TFs that regulate drought response, including NAC and WRKY TFs (Vermeirssen et al., 2014). This module is recognized by a TGACG-sequence-specific DNA-binding protein (TGA) transcription factor (Fode et al., 2008), which in our study showed higher initial expression in roots and leaves of CamB genotype. Other studies show that TGA TFs do not act alone in the induction of expression of their targets genes, but cooperate with other TFs, for example with SCARECROW-like proteins from the GRAS family. SCARECROW-like protein is a TGA transcription co-activator that regulates the expression of NAC032 gene in *Arabidopsis* (Fode et al., 2008). A gene encoding one of SCARECROW-like proteins (SCARECROW-like protein 33) had also higher initial expression in CamB leaves. Such observation points to the importance of this TGA–SCARECROW-like regulatory network in the survival of water deficit in barley and suggests that an interconnection of those two TFs is important for gene expression regulation, particularly in leaves.

Translation is another basic cellular process that is affected by drought. Its regulation relies on the action of translation initiation, elongation and termination factors, as well as on the proper assembly of ribosomes. In our study, higher initial expression of a gene for eIF3 subunit 6-interacting protein (eIF3-Int-6) and a gene encoding a putative eIF2 subunit 2 was found in roots and leaves of CamB genotype, respectively. eIF3-Int-6 protein has been found to interact with ribosomes, with the subunits of proteasome and Cop9 signalosome and to negatively regulate translation initiation (Paz-Aviram et al., 2008). Moreover, different eIF3 complexes may be present within the cell, as shown in fission yeast, and they are probably associated with the translation of different mRNAs (Zhou et al., 2005). Some eIF3 complexes may also promote translation of specific mRNAs, which contain upstream open reading frames (uORFs)–at least two codons present in 5′-leader sequences (Szamecz et al., 2008). uORFs are characteristic for about 20% of plant genes (Kochetov et al., 2002) and may be found in many genes involved in signal transduction or transcription regulation (Kawaguchi and Bailey-Serres, 2005; Rahmani et al., 2009). Their presence usually inhibits translation, but this process may be initiated under amino acid starvation and it is triggered by the phosphorylation of eIF2α, making the eIF2α-GTP–tRNAmet complex less available, resulting in the prolonged scanning of the 5′-leader by the 40S ribosomal subunit and the selection of other start codon, alternative to AUG (Morris and Geballe, 2000; Szamecz et al., 2008). Additionally, it was shown that the presence of uORFs in gene transcripts may have an impact on their differential regulation under dehydration stress in *Arabidopsis* leaves (Kawaguchi and Bailey-Serres, 2005). Our observation of higher expression of eIF3-Int-6 in roots and eIF2 subunit 2 in leaves may be related to the regulation of translation of uORF-containing transcripts in drought stress conditions.

Another gene encoding chloroplast elongation factor, EF-G, was also found to have a higher initial expression in CamB leaves. It functions in the translocation step of translation and in the recycling of ribosomes (Savelsbergh et al., 2009) and it may be required to sustain protein synthesis in chloroplasts under drought.

In addition to genes encoding translation controlling factors, a group of genes for ribosomal proteins may also be putatively involved in drought tolerance in barley. Three transcripts encoding chloroplast ribosomal proteins and one for 50S ribosomal protein L18 had higher initial expression in CamB leaves. The last one was also initially lower in CamB roots. Additionally, one gene for cytosolic 60S ribosomal protein L10a had lower initial expression in CamB. Higher expression of chloroplast ribosomal genes in leaves may simply be related to the higher demand for chloroplast proteins which are necessary to fulfill protein turnover in this organellum. Lower expression of one of the cytosolic ribosomal proteins may result from a more complicated regulatory mechanism related to the existence of many gene copies that encode ribosomal proteins variants in plants. It has been shown that their expression may differ depending on tissue, developmental stage or environmental conditions (Xue and Barna, 2012; Wang et al., 2013) and it has been proposed that such paralog-specific roles of ribosomal protein genes may form a “ribosomal code” influencing the overall gene expression regulation (Komili et al., 2007). There are also emerging evidences that specific ribosomal proteins may regulate translation initiation of transcripts with uORFs in plants (Nishimura et al., 2005; Szamecz et al., 2008).

#### Genes involved in signaling pathways

Several genes involved in phospholipid signaling pathway, which is already well recognized as important in stress response, have emerged as candidates for better drought tolerance in barley in our study. They include genes encoding phosphatidylinositol 4-kinase gamma 6, type IV inositol polyphosphate 5-phosphatase 11 (IP5P11), type I inositol 1,4,5-trisphosphate 5-phosphatase CVP2, diacylglycerol kinase 6, putative lipid phosphate phosphatase 3 and protein phosphatase 2C. All these genes play a role in signaling pathways *via* phosphatidylinositol phosphates and phosphatidic acid and are involved in ABA-mediated signal transduction (Wang et al., 2007; Paradis et al., 2011).

From this group, two genes drive special attention: up-regulated *IP5P11* and down-regulated *CVP2*. The function of *IP5P11* gene is not very well studied. It was shown, however, that its expression was stimulated by ABA and the IP5P11 enzyme hydrolyzes three types of inositol phosphates substrates [PtdIns(4,5)P_2_, PtdIns(3,5)P_2_, and PtdIns(3,4,5)P_3_], what may result in termination of signal transduction (Ercetin and Gillaspy, 2004). The second gene, *CPV2*, has been previously characterized as important for vascular tissue pattern formation in cotyledons (Carland and Nelson, 2004). The study of *cpv2* knock-out mutant in *Arabidopsis* showed that it accumulates more Ins(1,4,5)P_3_ than wild-type plants and is more sensitive to exogenous ABA, but does not show increased stress sensitivity (Carland and Nelson, 2004). Taking into consideration that different inositol polyphosphate 5-phosphatases have distinct substrate preferences (Carland and Nelson, 2004; Ercetin and Gillaspy, 2004), we may suppose that an interplay between expression levels of *IP5P11* and *CVP2* genes and resulting differences in the quantity of their dephosphorylation products are important for drought tolerance.

Provided that the above-mentioned genes were found to be important for effective drought response in both of studied organs, the majority of other genes involved in cell signaling was found to have higher initial expression only in roots of CamB genotype. They include gene homologous to a calcium-binding allergen Ole e 8, which has an EF-hand motive and plays a role in the Ca^2+^ signaling network and three genes from ABA signaling pathway. These are: probable 9-cis-epoxycarotenoid dioxygenase NCED5 involved in ABA synthesis (Frey et al., 2012); GEM-like protein 5, an ABA-responsive protein that binds various phospholipids, which is probably involved in ABA-signaling, (Mauri et al., 2016); and a putative ABA 8′-hydroxylase which inactivates ABA *via* its hydrolysis (Takeuchi et al., 2016). An interesting observation was the detection of higher initial expression of two genes with contradictory effects–ABA synthesis and ABA degradation. At the same time, literature data suggest that inhibition of ABA 8′-hydroxylase may increase drought tolerance, as was shown in *Arabidopsis* (Takeuchi et al., 2016). Our results indicate that a more sophisticated regulation of ABA content is required to successfully withstand drought stress and the accelerated degradation of ABA in roots, may be as important as its efficient synthesis. It is likely that turning on ABA responsive mechanisms within root tissues is regulated *via* ABA oscillation and an efficient turnover of ABA in roots may be required to set up a drought tolerance. Moreover, there are evidences that root system development under osmotic stress is driven be a crosstalk of ABA, cytokinin, ethylene and auxin, which all together form a network of competing activation-suppression mechanisms (Rowe et al., 2016), in which the fast ABA turnover may be an important element.

Ethylene signaling pathway was also represented in our study by the observation of a higher initial expression of a gene encoding EIN3-binding F-box protein 1 (EBF1) in CamB roots. This is a component of E3 ubiquitin ligase complexes that guide proteins to degradation. The target of EBF1 is the transcriptional activator EIN3, which regulates expression of ERF factors related to ethylene response pathways (Potuschak et al., 2003). Because ethylene, triggered by ABA, is known to inhibit root growth (Luo et al., 2014), degradation of EIN3 by EBF1 may be another mechanism that allows to sustain root elongation during the drought stress.

Another layer in the mechanisms of drought tolerance may be related to brassinosteroid (BR) synthesis. In roots of CamB genotype we found a higher initial expression of a *DET2* barley homolog that acts at relatively early steps in BR biosynthesis, accompanied with lower initial expression of a gene homologous to *CYP90D1*, which acts later in this pathway. Other studies showed that exogenous treatment with very low concentrations of brassinolide (BL) promotes root growth, but high concentrations of BL have inhibiting effects on this process (González-García et al., 2011). Additionally, the work of Ohnishi et al. (2006) suggests that CYP90D1 provides hydroxylation shortcuts that allow to omit some steps of BR synthesis in *Arabidopsis*. The observation of our study indicate that BR synthesis should be effectively initiated in roots, but CYP90D1 may act as a regulatory point, which slows down the BR synthesis, what may have a rate-limiting effect on this process.

The above-mentioned ubiquitin-mediated protein degradation is a process that may play broader role in drought tolerance in barley, not only in roots but also in leaves. Except of *EBF1* gene, four other genes related to this process (with POZ and MATH domain) had lower or higher initial expression observed in leaves of CamB genotype. Proteins with POZ and MATH domains may act as a substrate-specific adapter of an E3 ubiquitin-protein ligase complex. They have a broad substrate affinity and are responsible for proteasomal degradation of target proteins. They bind, for example, to HD-ZIP and ERF/AP2 TFs, and take part in ABA and ethylene signaling. They were also shown to be the receptors of salicylic acid and regulate the activity of TGA TFs in a dose-specific manner, which in turn regulate expression of their downstream targets (reviewed in Choi et al., 2014). Differential expression of genes encoding POZ domain proteins under drought stress in barley emphasize the importance of ubiquitin-mediated degradation of proteins as a regulatory mechanism of drought tolerance.

#### Genes controlling photosynthesis and plastid development

Among the genes regulating photosynthesis process, 12 have emerged in our study as candidates for better drought tolerance. Three belong to the chloroplast redox network and showed a higher initial expression in leaves of CamB genotype. One of them encodes thioredoxin reductase and the other two belong to ferredoxin family. Thioredoxin reductases are required for the reduction of oxidized thioredoxins using ferredoxin or NADPH as a reducing power (Nikkanen and Rintamäki, 2014). These chloroplast components are responsible for the redox homeostasis during photosynthesis, but were also shown to regulate starch, nitrogen and sulfur metabolism (Nikkanen and Rintamäki, 2014). Their primary role in drought tolerance may be attributed to the maintenance of electron transfer during photosynthesis, preventing overreduction of stroma proteins and providing the mechanism for the detoxification of oxidized chlorophyll (Hanke and Mulo, 2013). Another gene with similar expression pattern encodes chlororespiratory reduction 6 protein, which is involved in photosystem I cyclic electron transport and chlororespiration in higher plants. Similarly to thioredoxin system it may protect chloroplast from stromal overreduction (Munekage et al., 2004).

Two other genes that may be important for drought tolerance encode an *Arabidopsis* homolog of CURVATURE THYLAKOID1A protein, involved in the modification of thylakoid architecture by inducing membrane curvature (Armbruster et al., 2013) and GUN4, a regulator of chlorophyll synthesis and intracellular signaling (Larkin et al., 2003). Both had higher initial expression in CamB leaves under optimal water supply, indicating that chloroplast biogenesis is generally more efficient in drought tolerant genotype. This is in agreement with previous observations of CamB chloroplast morphology under drought, which showed that these organella were still well developed and were not reduced in number after the application of stress (Filek et al., 2016).

The other three genes from this group encode different subunits of photosystem I reaction center (PSI). A gene for subunit III (PsaF) had a higher initial expression in leaves of CamB. A gene for subunit XI (PsaL) had an opposite expression pattern–the lower initial expression in CamB. A gene for subunit V (PsaG) had again a higher initial expression, noticed not only in leaves, but also in roots of CamB, combined with its up-regulation in Maresi in both organs under drought stress. Each of these subunits of PSI have a distinct function in leaf chloroplasts. PsaF is necessary for electron transfer from plastocyanin to reaction center P700 and for precise docking of plastocyanin to PSI (Farah et al., 1995). Data on the function of PsaL is rather limited, but it was shown in the study of cyanobacteria *Synechocystis* sp. that it is required for PSI trimer formation. Mutants lacking PsaL have a monomer structure of PSI, what is accompanied with a higher accumulation of myxoxanthophyll and zeaxanthin carotenoids (Kłodawska et al., 2015). Previous analysis showed that CamB was characterized by greater accumulation of carotenoids in chloroplasts than Maresi, what was correlated with an increase in carotenoid radicals (Filek et al., 2016). Down-regulation of *PsaL* gene expression observed in our study in barley may indicate that it serves as one of the driving mechanisms of specific carotenoid accumulation, what in turn may be used to protect chloroplasts from the excess of light energy.

The most intriguing result was the observation of up-regulation of a gene for PsaG not only in leaves, but also in roots of both barley genotypes. This subunit is probably necessary to stabilize the core of PSI (Varotto et al., 2002). Its role in drought response or tolerance in roots may only be speculated, but, as suggested earlier, it may participate in ROS scavenging mechanism in roots. Such idea is supported by the observation of drought-induced up-regulation of chloroplast thioredoxin reductase gene in roots of both barley genotypes and the ferredoxin encoding gene in roots of Maresi cultivar. All these genes with ROS scavenging function may be a part of an “anti-oxidative center” in roots.

There were two other genes related to dark reactions of photosynthesis, which had a higher initial expression in roots of CamB. They encode phosphoribulokinase (PRK) and Calvin cycle protein CP12-1. PRK plays a role in the regulation of sugar flow through the Calvin cycle and has been shown to be down-regulated in leaves of C4 perennial grass species by drought and ABA, but up-regulated by light (Hu et al., 2012). The Calvin cycle protein CP12 acts as a linker in the assembly of a core complex of PRK/glyceraldehyde-3-phosphate dehydrogenase (GAPDH) and regulates both GAPDH and PRK during darkness in photosynthetic tissues (Singh et al., 2008). It was also shown that the activity of GAPDH and PRK proteins is regulated by thioredoxins and probably depends on the accumulation of reduced thioredoxins and metabolites in the chloroplast stroma (Marri et al., 2005). Importantly, the study of *Arabidopsis CP12* genes have shown that two of three genes, namely, *CP12-1* and *CP12-3* are expressed also in roots. *CP12-1* was localized in root tips and *CP12-3* throughout the root tissues (Singh et al., 2008). These findings and the observations of our study indicate that CP12 proteins, together with their target PRK may play a wider role in non-photosynthetic tissues and may be an important player in the maintenance of redox homeostasis in roots under drought stress.

A gene with a higher initial expression in roots of CamB, which encodes a plastid-lipid associated protein PAP was also found in this group. It belongs to fibrillin family, which is known to accumulate in fibrillar-type chromoplasts of ripening pepper fruits, and was also found in leaf chloroplasts from *Solanaceae* plants under stress conditions (Langenkamper et al., 2001). This type of plastids accumulate carotenoids and may represent another element of ROS scavenging system operating in roots, or play a role in root developmental processes under drought stress. It is known that carotenoids are the precursors in ABA synthesis (van Norman et al., 2014) and their accumulation in roots may be required for ABA-driven drought response.

#### Genes encoding enzymes involved in glycolysis, fermentation and pentose phosphate pathway

Respiratory pathway is another basic cellular process which is important in shaping plant response to drought stress and establishing tolerance to water deficit. In order to tolerate this stress, the plant needs to support itself with sufficient energy supply. It seems, however, that a move toward anaerobic or alternative respiratory pathways, especially in roots, may be profitable to cope with the stress.

Glycolysis is the most basic process of carbohydrate metabolism and several genes involved in this pathway showed a higher initial expression in CamB genotype. They include sequences encoding hexokinase, pyrophosphate:fructose 6-phosphate 1-phosphotransferase, glyceraldehyde-3-phosphate dehydrogenase 1, phosphoglycerate kinase and α-enolase, which are involved in subsequent steps of glucose metabolism. Similar enzymes were found to be more abundant in a proteomic study of grapevine under water deficit (Cramer et al., 2013) or in the transcriptome of annual ryegrass after drought stress (Pan et al., 2016). These findings points to the importance of efficient glycolysis in drought tolerance.

On the other hand, a gene for ATP-dependent 6-phosphofructokinase 4 (PFK4) was downregulated by drought in Maresi and had already a reduced expression in CamB leaves under control conditions. This enzyme is localized in chloroplasts and participates in plastidal glycolysis. It has been suggested that it plays a role in starch breakdown leading to the generation of metabolites for biosynthetic processes in dark-adapted or non-photosynthetic plastids, but it may also be inactivated in a light-dependent manner to avoid breakdown of photosynthesis products (Mustroph et al., 2013). If this hypothesis is true, it may support a conclusion that the lower expression of *PFK4* gene favors a conservation of energy resources and is advantageous for tolerance of prolonged drought.

Additionally, two different genes annotated as pyruvate kinase (PK) had either higher initial expression in leaves or lower expression in roots of CamB. Pyruvate kinases catalyze the final step of glycolysis, the production of pyruvate, which has been demonstrated to regulate the overall rate of glycolysis and respiration (Podesta and Plaxton, 1991). Stress-influenced down-regulation of these enzymes in leaves was noticed in several previous studies (Cramer et al., 2013; Yao and Wu, 2016), but drought-induced up-regulation was also previously found (Rodrigues et al., 2009; Li et al., 2016). These contradictory observations may be explained by the existence of different isoforms of pyruvate kinases in plants, which have different tissue specificity and cellular location. The increased activity of some isoforms in low oxygen stress was shown in castor oil, probably in order to compensate for the decreasing levels of ATP (Podesta and Plaxton, 1991; Turner et al., 2005). In leaves under drought stress, when the stomata are closed, the level of oxygen is reduced, thus the increase of PK expression in leaves may be one of the mechanisms to provide the energy. There is also other possibility, related to the existence of an alternative respiratory pathways in plants, which allow to circumvent enzyme-specific limitation to the glycolysis (reviewed in van Dongen et al., 2011). We may speculate that the reduced expression of other *PK* gene in roots may lead to such move of root respiration toward an alternative pathway. An indirect support of such possibility is also suggested by our observation of a higher initial expression of a gene encoding glucose-6-phosphate 1-dehydrogenase in roots of the tolerant genotype. This enzyme is involved in the pentose phosphate pathway, a parallel process to glycolysis, which mainly serves to generate NADPH, necessary for synthesis of such compounds as nucleotides and amino acids or to maintain redox potential in tissues exposed to oxidative stress (Kruger and von Schaewen, 2003). Thus, a higher activity of this biochemical pathway in roots may favor better adaptation to drought stress conditions.

A higher initial expression of genes involved in fermentation processes, encoding probable pyruvate decarboxylase (PDC) and alcohol dehydrogenase 1 (ADH1), may be another layer of better drought tolerance observed in CamB genotype, active mostly in roots. *PDC* and *ADH* genes are known to be induced during hypoxia conditions (Kürsteiner et al., 2003) and it is suggested that ADH plays an important role of hypoxia acclimation specifically in roots (Ellis et al., 1999).

#### Genes involved in cytoskeleton formation

It has been suggested that actin filaments (AFs) may participate in drought tolerance, as they form a network connecting cell walls, plasma membranes and cellular compartments. Through this physical connections they may act as osmotic sensors, because a decrease of turgor changes the compression of AFs, what in turn may be recognized by the cell as a signal to switch on expression of drought-responsive genes (Huang et al., 2012; Sniegowska-Swierk et al., 2015, 2016). Previous studies of AFs in CamB and Maresi genotypes showed that these genotypes differ in actin content and AF organization, both in optimal water conditions and after leaf desiccation (Sniegowska-Swierk et al., 2015). This observation was accompanied with an increase of *actin 11* (*ACT11*) expression in leaves of Maresi and *actin depolymerizing factor 3* (*ADF3*) expression in leaves of both genotypes (Sniegowska-Swierk et al., 2016). Our transcriptome analysis is consistent with these previous data, showing an increase in the expression of *ADF3* gene in both genotypes and both organs under drought stress. Importantly, *ADF3* gene had also higher than in Maresi initial expression in CamB leaves under control conditions, what may be an evidence that ADF3 is an important element of cellular signal transduction pathway under drought. Additionally, higher initial expression in roots of CamB was found for genes encoding actin and a probable LIM domain-containing serine/threonine-protein kinase. Expression pattern of these genes in roots–the first organ that perceives water decrease, supports the hypothesis of the role of AFs as the osmotic sensors.

An interesting and novel gene that may have an impact on drought tolerance in barley encodes a homolog of serine/threonine-protein kinase ULK4, whose expression was initially lower in CamB roots than in Maresi. There is very limited information about ULK4 proteins in plants, but studies of human or yeast ULKs show that they regulate autophagy and mediate remodeling of microtubule cytoskeleton (Mizushima, 2010; Lang et al., 2014). Autophagy is a mechanism that allows to cope with nutrient starvation upon stresses and enable cell survival, but there is a need of precise regulation between autophagy and maintenance of growth, in which ULK proteins are important players (Jung et al., 2010). The observed decrease of *ULK4* expression in roots of drought tolerant genotype may serve as a protecting mechanism against excessive cellular degradation upon drought stress, when a plant is exposed to temporal starvation caused by a lower availability of assimilates in the stress conditions.

#### Genes involved in vesicle transport

Drought tolerance rely also on an efficient vesicular transport, including secretory pathways and protein recycling *via* endosomal trafficking. Results from our study suggest that enhanced expression of genes encoding homologs of a transmembrane emp24 domain-containing protein 3 and a charged multivesicular body protein 1b (CHMP1b) may be important for efficient drought response in roots. The first protein that belongs to p24 family, is known in plants to cycle between endoplasmic reticulum (ER) and Golgi, and functions probably in the early secretory pathway as a cargo adaptor/receptor that specifically interacts with cargo molecules (Langhans et al., 2008). The second protein plays a role in endosomal sorting and is required for the dissociation of transport complex III (ESCRT-III) from the endosomal membrane after the recognition of ubiquitinated proteins by the endosomal complexes (Spitzer et al., 2009). Moreover, it has been shown in the study of *Arabidopsis* that CHMP1 proteins are involved in the transport of auxin carriers PIN1, PIN2, and AUX1 (Spitzer et al., 2009) what places this protein within the pathways connected with auxin signaling. Osmotic stress modulates the level of auxin transporters and their localization, as shows the study of *Arabidopsis*, where PIN1 level was reduced under stress in an ABA-dependent manner (Rowe et al., 2016). Thus, a higher level of *CHMP1b* gene expression may be another important factor of auxin signaling during the drought stress in roots.

#### Genes related to drought escape mechanisms

One of possible mechanisms established by plants to cope with drought stress is the adjustment of their flowering to the most optimal time of vegetative season, when the availability of water is not much depleted, yet. Indeed, the Syrian genotype CamB shows an early flowering phenotype, what was also confirmed by our transcriptome study. Five genes involved in the control of flowering time were found as initially expressed at the higher level in CamB genotype than in Maresi. Interestingly, in four cases this higher expression was found in both, roots and leaves. Two genes showed similarity to *Arabidopsis GIGANTEA* (*GI*) and other transcripts include *Arabidopsis* homologs of *MOTHER of FT and TF 1* (*MFT*), *BROTHER of FT and TFL 1* (*BFT*) and *Pseudo-response regulator 95* (*PRR95*). *GI* together with and *MFT* gene promote flowering in *Arabidopsis via* induction of *FLOWERING LOCUS T* (*FT*), and their expression is induced by ABA, what may explain accelerated flowering in response to drought stress (Riboni et al., 2013). On the other hand, *BFT* is a negative regulator of *FT* and flowering time, and was shown to delay flowering in response to salinity stress (Ryu et al., 2011). Its expression was also induced by ABA and was influenced by *GI* (Riboni et al., 2014). Our results suggest that all three genes: *GI, MFT*, and *BFT* may be involved in promotion of flowering which may allow drought escape, although *BFT* probably plays a modulating role in this process. Such possibility was also proposed by Riboni et al. (2014), who hypothesized that *BFT* may buffer *FT* activity in *Arabidopsis* and prevent a premature interruption of inflorescence development.

The last gene from this group detected in our study, *PRR95*, is a member of a pseudo-response regulators family-the components of the circadian clock. Experiments in *Arabidopsis* showed that PRR genes are expressed sequentially during the day, what results in circadian waves regulating endogenous circadian clock (Makino et al., 2001). In barley, several *PRR* genes were up-regulated in increasing temperature, but only *PRR95* was induced under long-day oscillating conditions (Ford et al., 2016). These data and our results implicate that *PRR95* gene may play a role in flowering time regulation in response to environmental stresses.

## Conclusions

To conclude, our global transcriptomic study shows that drought tolerance may result from stressed-like expression profile of many drought response genes, which is operating even before the occurrence of stress and makes the plant ready to respond to adverse environmental conditions. Mechanisms of drought sensing, that in a tolerant genotype are active already during normal water availability, allow to establish efficient drought response much faster than in a sensitive genotype, in which these mechanisms are turn on only after the occurrence of stress. We have analyzed the global transcriptome data with an intention to draw a broad picture of possible drought tolerance mechanisms. Our goal was to emphasize the connections between different genes, taking into account the interplay between their up- and down-regulation, together with the existence of several network connections between selected factors. The role of a portion of discussed genes in stress tolerance was already subjected to experimental verification in other species with the use of mutant or overexpression lines, although the analysis of their function was many times limited to leaves. Here we have found that some factors may also be important for drought sensing and stress response in barley roots. The predicted role of other genes in drought tolerance was outlined based on their biochemical function and their location in a network of drought-related biochemical or signaling pathways. We believe that the presented global transcriptome profiling of barley roots and leaves, together with its in-depth analysis will serve as a good resource for further exploration of molecular mechanisms, which turn plant metabolism to efficient drought response and build up the tolerance to environmental stresses.

## Author contributions

AJ, JK, and IS conceived of and designed the experiments. KŻ performed drought stress treatment of CamB and Maresi. AJ and MS collected plant material and performed RNA extractions. MK performed microarray data analysis. MS and KG run qPCR analysis of selected genes. AJ performed gene ontology enrichment analysis, data interpretation and wrote the manuscript. All authors discussed the results and commented on the manuscript.

### Conflict of interest statement

The authors declare that the research was conducted in the absence of any commercial or financial relationships that could be construed as a potential conflict of interest.
