# Supplementary material for: No Time to Waste: Transcriptome Study Reveals that Drought Tolerance in Barley May Be Attributed to Stressed-Like Expression Patterns that Exist before the Occurrence of Stress
Source: Front Plant Sci. 2018 Jan 9;8:2212. doi: 10.3389/fpls.2017.02212 (PMC5767312; doi:10.3389/fpls.2017.02212)
Supplement: Supplementary file 1 [file Image1.PDF]

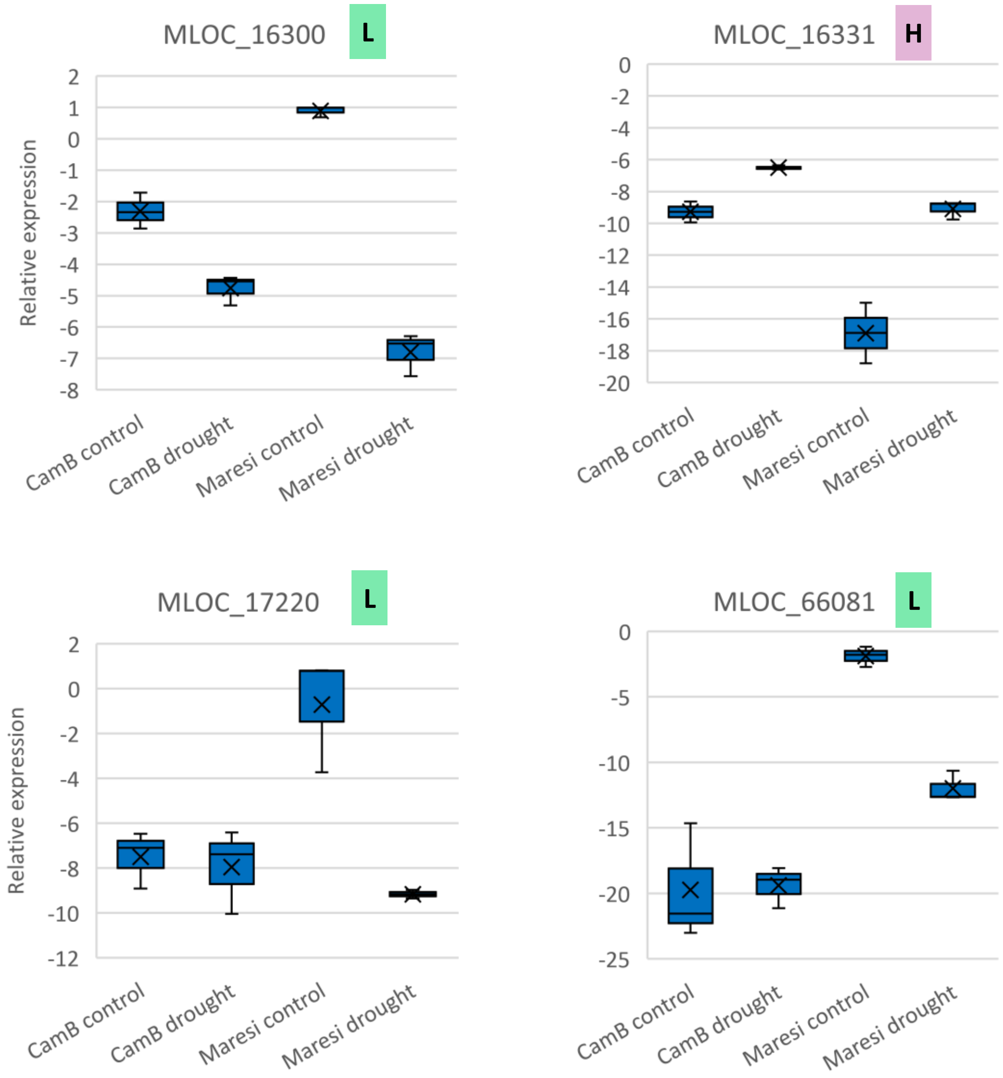

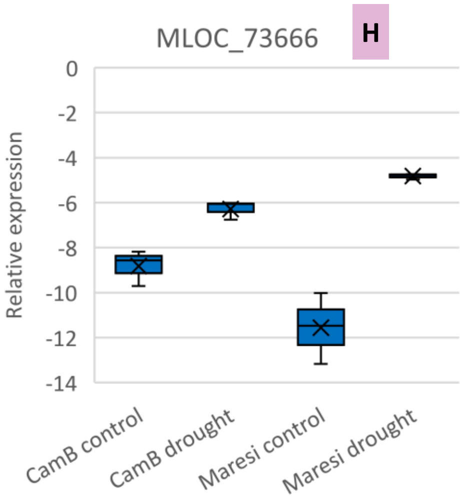

Supplementary Figure 1.

QPCR results of relative expression level of selected genes, which showed initially higher or lower expression in control conditions in CamB genotype versus Maresi cultivar in roots.

The relative expression level is shown as (40 – dCT); were dCt is the difference between Ct value of a gene of study and a reference gene.

A line within box plot represents median value, the asterisk is a mean value and the whiskers - minimum and maximum values.

H – higher initial expression in CamB roots, as measured by microarray analysis

L – lower initial expression in CamB roots, as measured by microarray analysis
