# Supplementary material for: No Time to Waste: Transcriptome Study Reveals that Drought Tolerance in Barley May Be Attributed to Stressed-Like Expression Patterns that Exist before the Occurrence of Stress
Source: Front Plant Sci. 2018 Jan 9;8:2212. doi: 10.3389/fpls.2017.02212 (PMC5767312; doi:10.3389/fpls.2017.02212)
Supplement: Supplementary file 4 [file Image4.PDF]

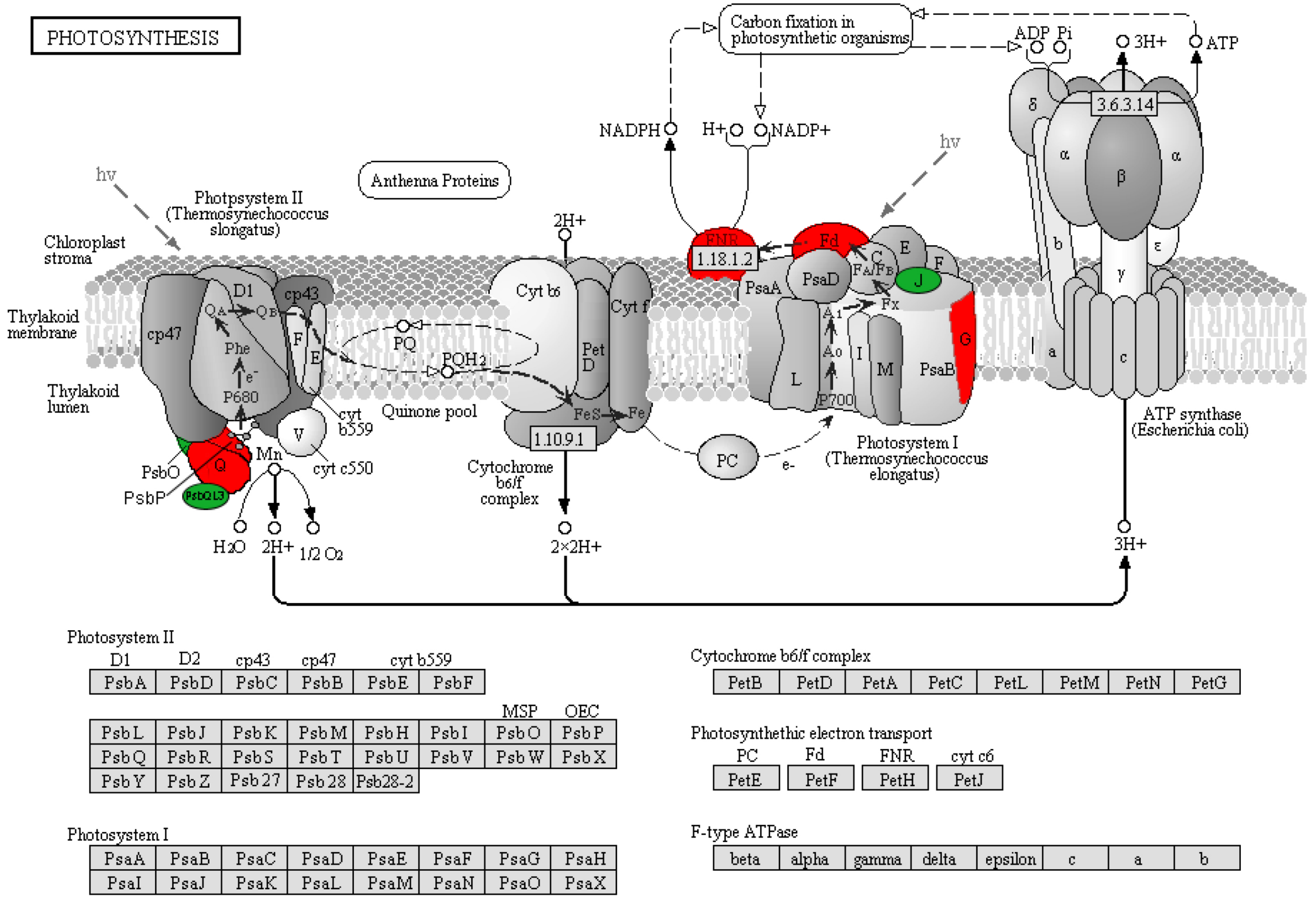

Supplementary Figure 4. Position of proteins encoded by genes differentially expressed in roots under drought within the photosystems I and II. Red – up-regulation under drought, green – down-regulation under drought. Abbreviations of DEGs from presented study: PsbO – Oxygen-evolving enhancer protein 1; PsbP – PsbP domain-containing protein; PsbQ – Oxygen-evolving enhancer protein 3; PsbQL3 – PsbQ-like protein 3; FNR – Ferredoxin-NADP reductase; Fd – Ferredoxin 1; J – Photosystem I reaction center subunit XI (PsaJ); G – Photosystem I reaction center subunit V (PsaG). The image was adapted from KEGG photosynthesis pathway (map00195), with modifications. For the remaining abbreviations, please see the KEGG database: http://www.genome.jp/kegg/kegg2.html).
