# Supplementary material for: No Time to Waste: Transcriptome Study Reveals that Drought Tolerance in Barley May Be Attributed to Stressed-Like Expression Patterns that Exist before the Occurrence of Stress
Source: Front Plant Sci. 2018 Jan 9;8:2212. doi: 10.3389/fpls.2017.02212 (PMC5767312; doi:10.3389/fpls.2017.02212)
Supplement: Supplementary file 5 [file Image5.PDF]

Light-harvesting chlorophyll protein complex

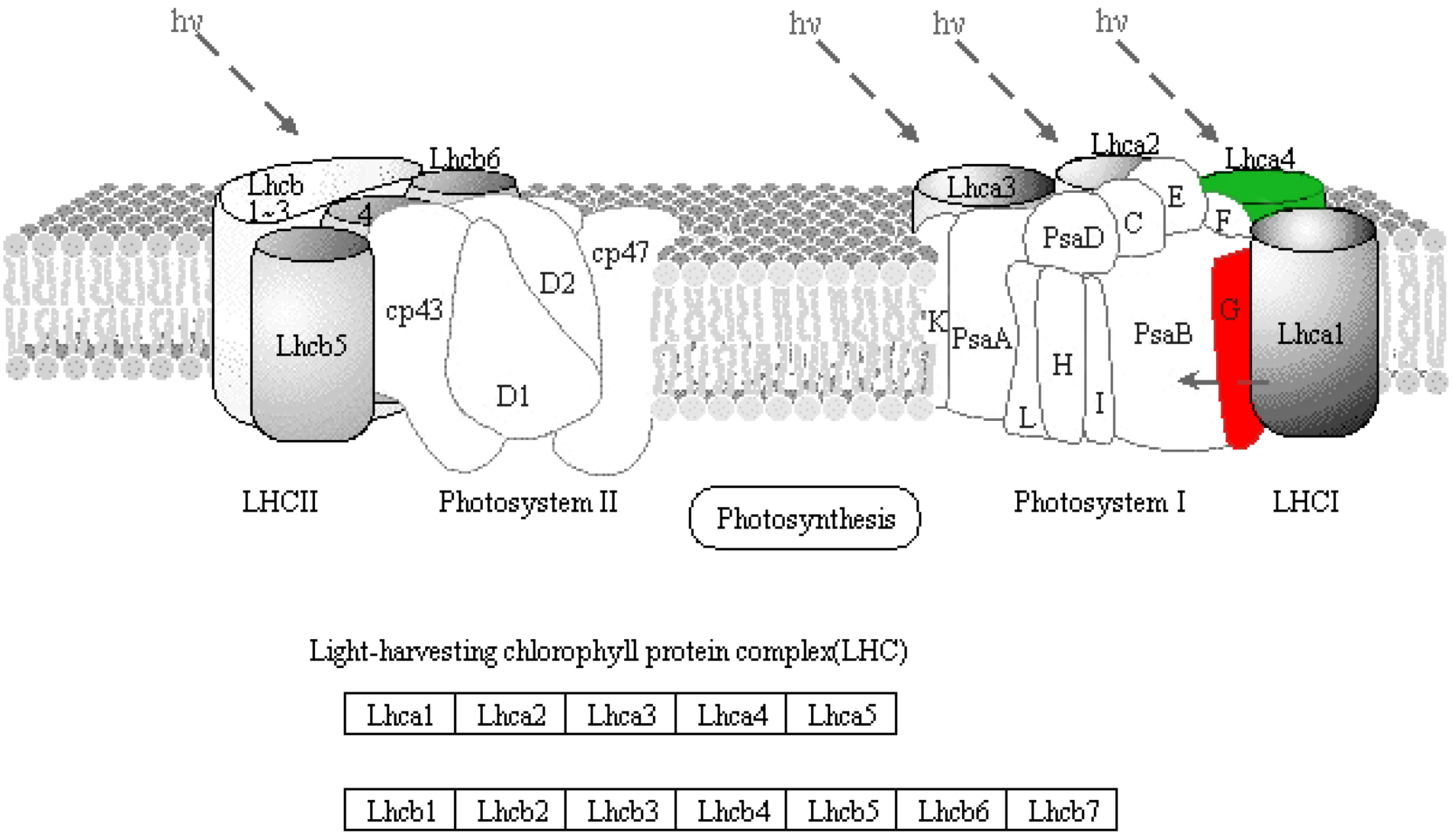

Supplementary Figure 5. Position of proteins encoded by genes differentially expressed in roots under drought within the antenna complexes and photosystem I. Red – up-regulation under drought, green – down-regulation under drought.
Abbreviations of DEGs from presented study:
G – Photosystem I reaction center subunit V (PsaG); Lhca4 – light-harvesting complex A4 (Chlorophyll b binding protein 1B-20). The image was adapted from KEGG photosynthesis pathway (map00196), with modifications. For the remaining abbreviations, please see the KEGG database: http://www.genome.jp/kegg/kegg2.html).
