# Supplementary material for: No Time to Waste: Transcriptome Study Reveals that Drought Tolerance in Barley May Be Attributed to Stressed-Like Expression Patterns that Exist before the Occurrence of Stress
Source: Front Plant Sci. 2018 Jan 9;8:2212. doi: 10.3389/fpls.2017.02212 (PMC5767312; doi:10.3389/fpls.2017.02212)
Supplement: Supplementary file 3 [file Image3.PDF]

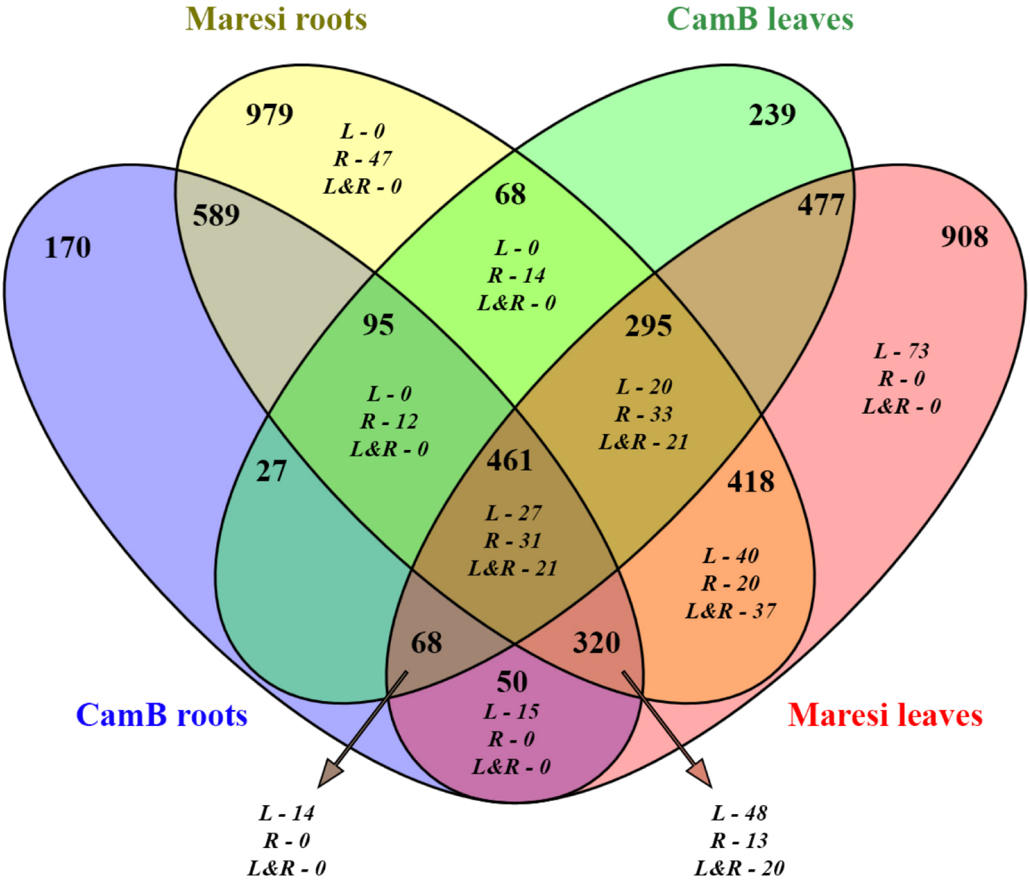

Supplementary Figure 3. Venn diagram showing a compilation of the number of differentially expressed genes after drought treatment in CamB and Maresi leaves and roots (DEGs) and the number of genes with higher or lower expression in control conditions in CamB genotype in the comparison to Maresi cultivar.

Bold and larger font – number of DEGs in particular combination of the genotype and organ;

Smaller font – number of genes with higher or lower expression in control conditions: L – only in leaves of CamB, R – only in roots of CamB, L&R – in leaves and roots of CamB in the comparison to Maresi.
